# Temporal and spatial variability of constitutive mixotroph abundance and proportion

**DOI:** 10.1093/femsec/fiae015

**Published:** 2024-02-02

**Authors:** Marcella Dobbertin da Costa, Rebecca J Gast, Nicole C Millette

**Affiliations:** Virginia Institute of Marine Science, William & Mary, 1370 Greate Rd., Gloucester Point, VA 23062, United States; Woods Hole Oceanographic Institution, 266 Woods Hole Rd, MS #32, Woods Hole, MA 02543, United States; Virginia Institute of Marine Science, William & Mary, 1370 Greate Rd., Gloucester Point, VA 23062, United States

**Keywords:** BrdU, community composition, environmental factors, estuaries, mixotrophs

## Abstract

Mixotrophic plankton can comprise a substantial portion of the plankton community compared to phytoplankton and zooplankton. However, there is a gap in the understanding of conditions that influence mixotroph prevalence and activity *in situ* because current methods often over- or underestimate mixotroph abundance. A labeled prey-tracer method was utilized to identify active mixotrophs present at two locations in a temperate estuary over a year. The tracer method was combined with light microscopy data to estimate active mixotroph abundance and proportion. This study estimated that actively grazing mixotrophic taxa were more abundant in the spring and autumn compared to summer. Dinoflagellates typically dominated the mixotrophic taxa except during autumn at the low salinity location when cryptophytes dominated. Further analysis suggested that active mixotroph abundances might not be only regulated by environmental conditions favorable to mixotrophy but, instead, environmental conditions favorable to different mixotrophs utilization of phagotrophy. By focusing on mixotrophic taxa that were identified to be actively grazing at time of sampling, this study provided a more nuanced estimation of mixotroph abundance, increasing the understanding of how mixotrophic abundance and proportion *in situ* are influenced by the planktonic community composition and environmental factors.

## Teaser

A novel method using labeled prey tracers in a temperate estuary showed that actively grazing mixotrophic plankton are more abundant in spring and autumn and their presence may depend on environmental conditions favorable to different mixotrophs utilization of phagotrophy.

## Introduction

The existence of mixotrophic plankton (organisms that combine photoautotrophy and phagotrophy) has long been recognized (Flynn et al. 2013). However, it has only been within the past decade that scientists have appreciated that mixotrophs can comprise a substantial portion of the plankton community (Flynn et al. 2013, Stoecker et al. 2017).While mixotrophs are known to be prevalent, research on them lags behind phytoplankton and zooplankton. Recently, several research priorities for mixotrophs were outlined, one of which was improved categorization of the biogeographical distribution of mixotrophs (Millette et al. 2023). Specifically, an understanding of the spatial and temporal patterns of mixotroph abundance and proportion relative to phytoplankton and zooplankton is needed. It is important to know when mixotrophs are expected to be a prominent component of the plankton community because their role in trophic transfer and biogeochemical cycling is distinct from phytoplankton and zooplankton (Mitra et al. 2014, Ward and Follows 2016, Larsson et al. 2022). For example, a modeling study demonstrated that a global plankton food-web composed 100% of mixotrophs increased carbon sequestration by 35% compared to a food-web with no mixotrophs (Ward and Follows 2016). Unfortunately, without basic knowledge of what proportion of the plankton community is mixotrophic, it is impossible to assess the accuracy of this result. An important first step in predicting when and where mixotrophs are an important part of the plankton food web is to understand the spatial and temporal variability of mixotroph abundance and the environmental factors associated with their variability.

However, there is presently a methodological limitation in the ability to differentiate mixotrophs from phytoplankton and zooplankton in field studies (Millette et al. 2018), which limits our ability to study the biogeographical distribution of mixotrophs. For example, mixotrophs are typically grouped with phytoplankton due to the use of photopigments (chlorophyll *a*) as an indicator of photosynthetic capability. Furthermore, grazing activity is often attributed to only zooplankton because it is indirectly measured by looking at the change in prey concentration, rather than identifying organisms doing the grazing (Millette et al. 2018). Given how prevalent mixotroph taxa can be in the plankton community (Domaizon et al. 2003, Unrein et al. 2007, Millette et al. 2021, Li et al. 2023), research needs to transition toward identifying and studying mixotrophs as their own plankton group(s), apart from phytoplankton and zooplankton.

The ability to estimate mixotroph abundance and activity *in situ* is hindered by the limitations of current popular methods (Anderson et al. 2017, Li et al. 2021). Active mixotroph abundance is typically estimated with fluorescently labeled bacteria or fluorescent microspheres (Arenovski et al. 1995, Sanders et al. 2000, Czypionka et al. 2011, Anderson et al. 2017, Millette et al. 2017, Sato et al. 2017, Gast et al. 2018, Li et al. 2023). These methods work by estimating the abundance of cells that consume fluorescently labeled material and contain chloroplasts (González 1999). However, it has been demonstrated that this approach chronically *underestimates* mixotroph abundance (Anderson et al. 2017, Li et al. 2021) because these experiments have a short incubation period (< 2 h) that likely does not capture all grazing activity. Furthermore, certain species might be biased against fluorescently labeled particles, while others may be overly biased towards them (Sanders and Gast 2012, Wilken et al. 2019). Recent studies have also attempted to estimate mixotroph abundance in publicly available microscopy datasets via taxonomy (Haraguchi et al. 2018, Leles et al. 2019, Cesar-Ribeiro et al. 2020, Schneider et al. 2020, Li et al. 2023, Mitra et al. 2023). This approach uses available microscopy-based taxonomic data to estimate the abundance and proportion of potential mixotrophs, plankton that have been found to be capable of photoautotrophy and phagotrophy in previous peer-reviewed studies, over large temporal and spatial scales. However, this method is likely an *overestimation* of the presence of mixotrophs, since it is not known if a species is utilizing mixotrophy in any given sample or location.

Recently, bromodeoxyuridine (BrdU) has been utilized to identify actively grazing mixotrophic taxa in field samples (Fay et al. 2013, Millette et al. 2021). This method targets constitutive mixotrophs (CMs), organisms that actively synthesize and maintain their own chloroplasts, that ingest bacteria (Ghyoot et al. 2017). While this approach does not capture all possible mixotrophs present in a system, it provides taxonomic information on the CMs actively ingesting bacteria at any given time, which are an important and often dominant type of mixotroph (Jeong et al. 2010, Edwards 2019, Faure et al. 2019). In studies using BrdU-labeled prey, the assumptions are that mixotrophs will ingest and digest the prey, and then incorporate the labeled nucleotides in their own DNA during replication. Amplicon sequencing of selectively recovered DNA allows identification of active mixotrophs in the phototrophic taxa. Those mixotrophs can be matched with taxa identified in microscopy samples to estimate the abundance and proportion of mixotrophs (Millette et al. 2021). So, while this approach does not capture the full mixotrophic community, it does capture the majority of CMs in microscopy samples. A recent study by Millette et al. (2021) utilized the BrdU method to identify and quantify CMs in microscopy samples from Waquoit Bay, MA and suggested that CMs could account for over 90% of chloroplast containing plankton in a sample. However, BrdU experiments were only conducted for six of the 23 sample dates, so most of the conclusive results were based on potential CM abundance, rather than which taxa were actively grazing in each sample. Millette et al. (2021) demonstrated that estimating CM abundance this way was likely an overestimation because the presence of a mixotroph does not guarantee that a mixotroph is using its alternate nutrient mode (grazing). However, if the BrdU method was utilized every time a microscopy sample is collected, then the abundance of mixotrophs actively grazing in those samples could be estimated.

In this study, BrdU experiments were conducted for all sampling dates and used to constrain estimates of CM abundance in microscopy samples to taxa actively grazing. This made it possible to taxonomically identify and estimate CM abundances to assess the variability of CMs that ingest bacteria within a temperate estuary across a whole year. The goal was to investigate the biotic and abiotic factors associated with the temporal and spatial variability of active CMs identifiable though microscopy. To accomplish this goal, there were three objectives: (1) to develop lists of CMs grazing on bacteria in two distinct parts of the York River Estuary, Chesapeake Bay, USA, (2) compare the estimations of potential versus active CM abundance and proportion, and (3) use the active CM data to investigate the biotic and abiotic factors associated with the spatial and temporal variability of CM abundance and proportion. This study identified over 130 mixotroph taxa using the BrdU method that were matched up with seven taxa identified in microscopy samples. Results suggested that while CM abundance was related to environmental conditions, the abundance of active CMs was also related to the taxa present.

## Methodology

### Stations

**Figure 1.**
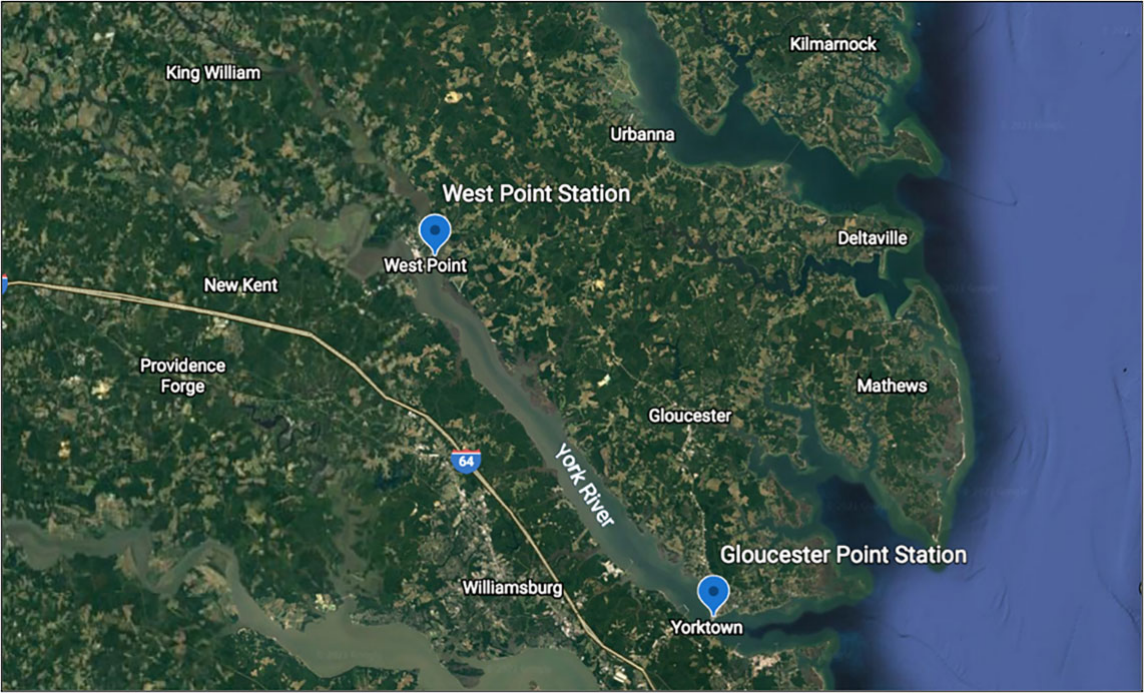
Map of location of fieldwork, with the two different sampling stations across the York River, VA marked with blue dot. WP Station—WP Fishing Pier and GP Station—GP Fishing Pier.

In total, two stations were sampled twice a month over 1 year in the York River Estuary, Virginia, USA; West Point (WP) and Gloucester Point (GP; Fig. 1). WP is located up-estuary, near one of the two major freshwater sources to the York River (Mattaponi River) at the WP fishing pier. GP is located closer to the mouth of the estuary, where the river flows into Chesapeake Bay, at the GP fishing pier. The WP station is characterized by low salinity (oligo- to mesohaline) with high nutrient concentrations and high turbidity, as it is located near the estuarine turbidity maximum (Friedrichs 2009, Reay 2009). The GP station is characterized by high salinity (meso- to polyhaline) with low nutrient concentrations and high turbidity compared to WP (Friedrichs 2009, Reay 2009).

### Environmental data

All sampling occurred on an incoming tide, no later than 1 h before high tide. WP was always sampled first, followed by GP. A YSI EXO1 sonde (Xylem Inc.) was used to conduct a vertical profile of temperature (°C), salinity, and turbidity (FNU: Formazin Nephelometric Unit). The data points for each profile were averaged for every 0.1 m and the averaged data points at 0.5 m were used for analyses. A LI-1400 (2$\pi $ quantum sensors; Deck: LI-190SA and Water: LI-192SA) from LI-COR was used to conduct an irradiance profile of the water column and calculate the light attenuation coefficient (k_d_) at a depth of 0.5 m.

A 5-l Niskin bottle (General Oceanics) was used to collect water from just below the surface. Water for nutrient analysis (30 ml of 0.45 µm-filtered water) was collected in two 20 ml acid-washed plastic vials. Once in the laboratory, no more than 2 h after samples were collected, the nutrient samples were frozen at −20°C until analysis for ammonia (NH_3_), nitrate + nitrite (NO_x_), phosphate (PO_4_^3−^), and silica (SiO_2_) (µM) using a Skalar Auto Analyzer in the Virginia Institute of Marine Science Analytical Services Center (U.S. EPA 1993a, 1993b, 1993c, 1997). Water for chlorophyll *a* analysis was collected in three 1 l clear Nalgene bottles. The bottles were kept in a dark cooler until they were brought back to the laboratory. To measure chlorophyll *a* concentrations, 40–150 ml of the water collected was filtered onto 25 mm GF/Fs. The filters were resuspended in 7 ml of 90% acetone for 24 h and placed in the freezer at −20°C. After 24 h, the samples were read on a Turner Designs 10-AU fluorometer for chlorophyll *a* concentrations before and after acidification with 10% HCl (Arar and Collins 1997).

### Microscopy

Additional water from the 5-l Niskin cast was collected in three 500 ml Nalgene amber bottles, immediately preserved with 5% Lugol’s solution, and sealed with electrical tape. The Lugol’s samples were concentrated in the laboratory by settling for 24 h in a 500-ml beaker and then gently pipetting liquid off the top to reduce the total volume to ∼50 ml. Chloroplast containing plankton genera/species in these samples were identified and enumerated (cells ml^−1^) with a Zeiss Axio Imager.A2 microscope at 400x magnification on a Sedgewick rafter slide (Sherr and Sherr 1993). A minimum of 300 cells were counted per sample.

### BrdU-labeled bacterial ingestion experiments

To identify actively grazing CMs present at each station on each sampling date, incubation experiments were conducted using BrdU-labeled bacteria as prey. Two cultures of *Photobacterium angustum* (heterotrophic bacteria) were grown in yeast extract 10 days before sampling. BrdU (800 µM final concentration) was then added to one of the bacterial cultures (+BrdU) 3 days before sampling (Millette et al. 2021). The BrdU was washed off the culture the day before sampling by centrifuging at 3000 r m^−1^ for 10 min at 4°C. The supernatant was removed without disturbing the bacterial pellet. A volume of 10 ml of sterile 1x phosphate-buffered saline (PBS) was used to resuspend pellets and they were centrifuged again. This was repeated three times and after the final wash, bacterial pellets were resuspended in a total of 20 ml sterile 1x PBS and cell concentration determined using hemocytometer. The bacterial culture without BrdU (−BrdU) was also “washed” three times to ensure both cultures were treated the same. The water collected from the Niskin cast was used to set up triplicate incubations for +BrdU and −BrdU bacterial additions. A volume of 250 ml of water was placed into a 250-ml clear Nalgene bottle and bacteria was added to a final concentration of 10^6^ cells ml^−1^. The bottles were then incubated for 24 h in one-layer mesh bags in the York River Estuary, near the Virginia Institute of Marine Science. At the end of the experiment, all water from each incubation bottle was collected onto 47 mm 3-µm Isopore filters and stored at −20°C until analyzed.

### Immunoprecipitation of ±BrdU DNA and sequence analysis

Nucleic acids were extracted for all samples following the hot detergent method reported by Gast et al. (2004) using 200 µl of lysis buffer. Extracted material from +BrdU incubations then went through an immunoprecipitation process to recover DNA that had incorporated BrdU. Nucleic acids recovered from the −BrdU incubation were not immunoprecipitated but used directly for amplicon PCR. The first step in the immunoprecipitation process involved preparing an antibody mix and a magnetic bead mix that were blocked with denatured bacterial DNA to help prevent nonspecific binding. A total of 300 ng of *P. angustum* DNA per sample (10 ul per reaction) was denatured for 10 min at 95°C, the tubes were transferred to an ice bath for 2 min, and then cold PBS–BSA [1 mg acetylated bovine serum albumin (BSA) per ml 1x PBS] was added to bring to a final volume of 30 µl per reaction. The denatured bacterial DNA was used to block the antibodies (anti-BrdU B44; BD Biosciences 347 580; 1/10 dilution: 2.5 ng µl^−1^; 10 µl each for each reaction) and the beads (Dynabeads M-280 Sheep antimouse magnetic beads; Invitrogen 11201D; 10 µl beads + 10 µl of PBS–BSA). The denatured bacterial DNA was mixed with same volume of antibody or beads in a 1.5-ml microfuge tube and incubated at 4°C on a rotating mixer for 1 h (antibody mix) or overnight (bead mix).

BrdU-labeled DNA samples from the experiments (dilution of 300 ng in 10 ul) were denatured at 95°C for 10 min, placed on ice for 2 min, and then 10 µl of cold PBS–BSA was added. Each denatured BrdU DNA sample was then combined with 20 µl of the blocked antibody mix and incubated overnight at 4°C on a rotating mixer. This step allowed for BrdU-labeled DNA to bind to the anti-BrdU mouse antibody. The following day 40 µl of blocked beads were added to each antibody sample and incubated for at least 1 h at 4°C on a rotating mixer to allow the BrdU-labeled DNA bound to the mouse antibody to bind to the beads. To remove non-BrdU-labeled DNA from the antibody/bead mixture, tubes were transferred to a magnet (DynaMag^TM^-2, Invitrogen 12321D) for about 5 min. Once the solution was clear, it was removed and discarded, and the beads were fully resuspended in 1 ml of washing buffer (PBS with 0.05% Tween 20). The solution was allowed to clear on the magnet, followed by removal of liquid without bead pellet disruption. This step was repeated three times. Then the beads were resuspended in 0.5 ml of washing buffer, the solution allowed to clear on the magnet, followed by removal of liquid without bead pellet disruption, repeated four times. To recover BrdU-labeled DNA from the beads, 100 µl of elution buffer (5 mM BrdU in PBS; Sigma B9285-250 mg) was added to each sample and incubated at 65°C for 20 min and then at room temperature for 15 min. The solution was allowed to clear on the magnet and the liquid transferred to a new tube for ethanol precipitation (90 µl 100% isopropanol, 35 µl 3 M NaCl, 100 µl elution) over night at −20°C. After centrifugation, the DNA pellet was allowed to dry for several minutes before resuspending in 10 µl PCR water.

Amplicons for −BrdU DNA and immunoprecipitated +BrdU DNA were generated through PCR amplification of the V4 region of the 18S ribosomal gene using primers V418SF (5′ [TCGTCGGCAGCGTCAGATGTGTATAAGAGACAG] CCAGCASCYGCGGTAATTCC) and V418SR (5′ [GTCTCGTGGGCTCGGAGATGTGTATAAGAGACAG] ACTTTCGTTCTTGATYRATGA), described in Piredda et al. (2017) and modified to include 5′ adapter sequences (in square brackets) for Illumina MiSeq. Each sample was amplified in triplicates using up to 3 µl template DNA, 1.25 units GoTaq Flexi DNA polymerase, 2 mM MgCl_2_, 2 µl 2.5 µM dNTPs, and 2.5 µl 10x reaction buffer (25 µl total volume) with the conditions: 95°C for 8 min; 35 cycles of 95°C for 30 s, 58°C for 30 s, 72°C for 90 s; 72°C for 5 min; 4°C hold. Some of the -BrdU samples needed to be diluted in order to be amplified (Table S2, Supporting Information). Amplicons were sent to the Rhode Island Institutional Development Award (IDeA) Network of Biomedical Research Excellence Molecular Informatics Core for library preparation and Illumina MiSeq (300 bp paired end; 600 cycle kit V2) sequencing.

QIIME2 was used to demultiplex, denoise, remove chimeras, and quality control the Illumina data. Amplicon sequence variants (ASVs) were grouped at 100% identity and taxonomy assigned using the Silva 132 database. ASVs identified as bacteria, metazoan, fungi, and macroalgae were removed from the analyzed dataset, as were those that occurred only once (singletons). For each experiment, taxa were identified as bacterivores based on comparison of tag sequences between +BrdU and −BrdU samples. ASV abundances were converted to a percentage of the total tags for each sample, and the average of each −BrdU ASV was subtracted from the average of the corresponding +BrdU ASV. An ASV was considered a bacterivore if the subtracted value was positive and > 0.1% of the average total amplicon abundance, suggesting that this ASV was present at a higher proportion in the +BrdU samples. Bacterivores identified as taxa containing chloroplasts were then considered actively grazing CMs (Fay et al. 2013, Millette et al. 2021). The use of this conservative approach was to represent the more abundant amplicons in the datasets, balance the effect of variability between incubation replicates and reduce the influence of nonspecific recovery of extremely abundant DNA (e.g. from diatoms; Millette et al. 2021).

### Potential and active CM abundance and proportion

The abundance of potential CMs was calculated by summing the abundance of genera present in each microscopy sample that had been identified as a CM in the BrdU experiments for any sampling date at a specific location. This calculation reflects the abundance of plankton with the ability to engage in mixotrophy but not whether they were actively ingesting bacteria prey, or using their alternate nutrient mode, as done in Millette et al. (2021). To calculate *active* CM abundance, a qualitative identification of a microscopic taxon as an active CM was made if there were matches at the genus level for each sampling date at each station. This calculation reflects the abundance of CMs that were actively engaging in mixotrophy at the time the sample was collected. The proportion of potential and active CMs were calculated by dividing the total abundance of taxa identified as either a potential or active CM by the total phototroph abundance for that sample. Potential CM abundance and proportion were compared to active CM abundance and proportion to assess potential overestimations in the presence of CMs when not accounting for which taxa are actively ingesting. It was assumed that any CM taxa that was present but not identified to be actively ingesting bacteria when a sample was collected was only photosynthesizing. Further analysis on biotic and abiotic factors associated with the abundance and proportion of CMs was only done on active CMs.

### Analysis

Quasipoisson generalized linear models with an offset (GLMs) were applied for both stations to examine environmental conditions associated with the abundance of diatoms, dinoflagellates, cryptophytes, haptophytes, and active CMs. Quasibinomial GLMs with an offset were applied for both stations to examine environmental conditions associated with the proportion of active CMs. Triplicate samples were kept separate and sample dates with any missing data were removed from analysis (WP: *n* = 57, GP: *n* = 48). The independent variables (environmental data) included were temperature, salinity, turbidity, attenuation coefficient (K_d_), and nutrient concentrations (NO_x_, NH_3_, PO_4_^3−^, and SiO_2_). The offset term was the number of grids on the microscope slide that was counted for each sample. The independent variables for each model run were tested for collinearity using the “car” package in R with the variance inflation factor (VIF) function (Fox and Weisberg 2019 ). Any environmental variable that had a collinearity VIF value of 5 or greater (Zuur et al. 2010) was removed so that analyses were run only with independent variables that were not strongly related to each other. Any variables in the selected GLM with *P*-values less than .5 were considered to have some signal and it was reported whether this signal was positively or negatively related to the dependent variable. All GLM runs were conducted in R (version 3.6.3) using the built-in linear regression (glm) function.

## Results

### Environmental data

The average physical and environmental data collected from March 2021 to February 2022 are presented in Table 1. The average turbidity, temperature, and K_d_ were significantly higher at WP while the average salinity was significantly higher at GP. However, the majority of biological (chlorophyll *a* concentrations) and chemical (nutrient concentrations) data were not different between WP and GP. The only exception to this was NO_x_ concentration, which was significantly higher at WP compared to GP. Graphs of the unaveraged time series of measurements illustrate the variability of environmental factors within and between stations (Figures S1–S9, Supporting Information).

**Table 1.** Average (±SE) water quality data collected 24 times between March 2021 and February 2022 at two stations in the York River.

	Chl *a* (µg l^−1^)	Sal*	Turb (FNU)*	Temp ($^\circ $C)*	NH_3_ (µM)	NO_x_ (µM)*	PO_4_^3−^ (µM)	SiO_2_ (µM)	K_d_ (dB m^−1^)*
WP	18.8$ \pm $3.5	6.5$ \pm $0.7	19.4$ \pm $2.3	18.9$ \pm $2.1	6.0$ \pm $1.0	2.3$ \pm $0.3	0.14$ \pm $0.02	6.2$ \pm $0.9	3.8$ \pm $0.2
GP	13.0$ \pm $1.6	19.0$ \pm $0.4	4.8$ \pm $0.6	18.2$ \pm $2.0	4.2$ \pm $0.7	0.4$ \pm $0.2	0.09$ \pm $0.03	8.7$ \pm $1.7	1.5$ \pm $0.1

Chl *a*: chlorophyll *a*; Sal: salinity; Turb: turbidity; Temp: temperature; NH_3_: ammonia concentration; NO_x_: nitrate + nitrite concentration; PO_4_^3−^: phosphate concentration; SiO_2_: silica concentration; and K_d_: light attenuation coefficient. *Significant difference between the two stations (paired, 2-tailed *t*-test, *P* < .05).

### Microscopy-based phototroph abundance and major taxonomic groups

**Figure 2.**
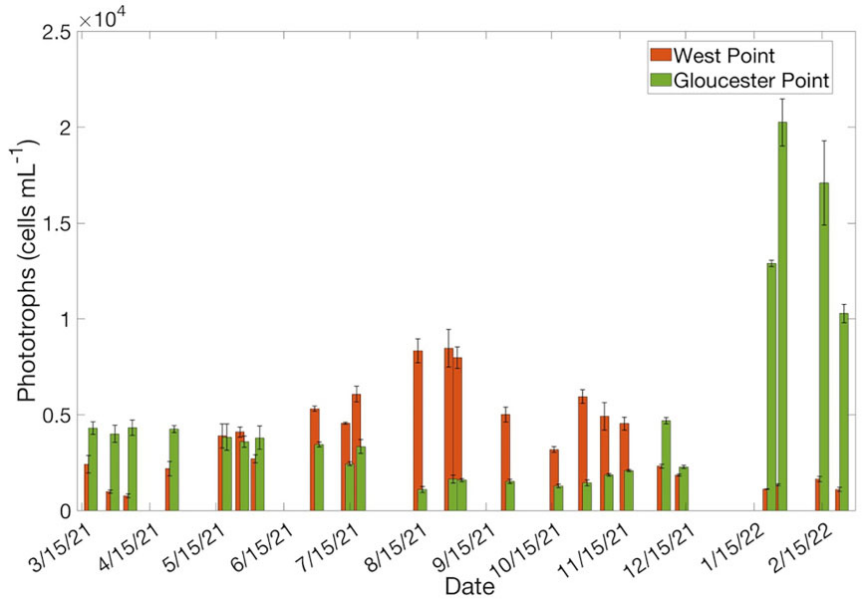
Total abundance of phototrophs (±SE) at WP and GP between March 2021 and February 2022. The *x*-axis represents a year-long timeline. Each pair of bars (WP and GP) represents a single sampling date along that timeline.

**Figure 3.**
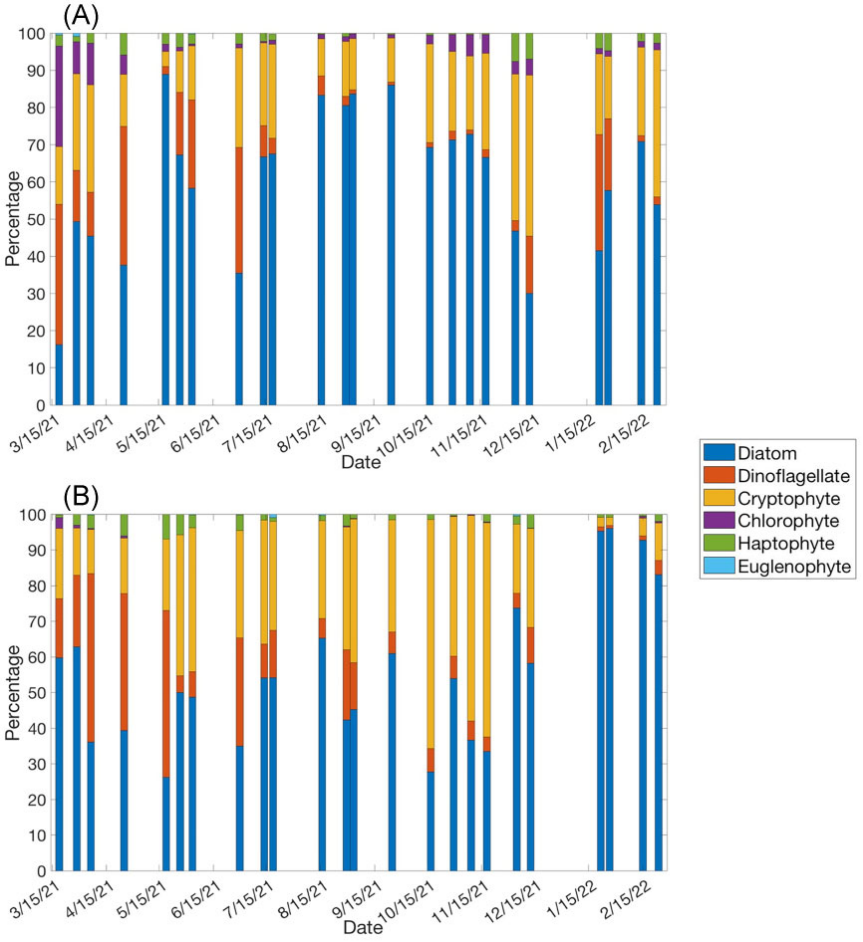
Proportion of total abundance that six plankton groups, identified through microscopy, accounted for at each sample date between March 2021 and February 2022 at (A) WP and (B) GP. The *x*-axis represents a year-long timeline. Each bar represents a sampling date.

The average (±SE) phototroph abundance based upon microscopy was not significantly different between WP (3777 ± 305 cells ml^−1^) and GP (4883 ± 367 cells ml^−1^, *P*-value = .43, *t*-test, Fig. 2). However, the temporal variability of phototroph abundance was high at both stations. The highest phototroph abundances at WP occurred during the late summer, during the months of August and September, and remained elevated through November (Fig. 2). At GP, phototroph abundances were highest during the winter, in the months of January and February due to a small diatom bloom dominated by *Skeletonema* spp. (Fig. 2). The proportion of major taxa groups present between stations and sampling dates was also highly variable. However, the general trend for both stations was dinoflagellates dominating during the spring, diatoms during the summer, cryptophytes during the autumn, and then diatoms again during the winter (Fig. 3A and B). At GP, the shift toward diatoms dominating again during the winter was specifically due to the aforementioned *Skeletonema* spp. bloom (Fig. 3B).

Based on results from the GLM analysis, at WP, diatom abundance was positively correlated to temperature, NH_3_ and SiO_2_, and negatively correlated to turbidity, NO_x_, and K_d_ (Table 2). Dinoflagellate abundance was positively correlated to K_d_ and negatively correlated to turbidity and NO_x_ (Table 2). Cryptophyte abundance was positively correlated to temperature, NH_3_, and SiO_2_, and negatively correlated to PO_4_^3−^ and K_d_ (Table 2). Haptophyte abundance was positively correlated to temperature, PO_4_^3−^, SiO_2_, and K_d_, and negatively correlated to NH_3_ (Table 2). At GP, diatom abundance was positively correlated to salinity, SiO_2_, and K_d_, and was negatively correlated to temperature, NO_x_, and PO_4_^3−^ (Table 2). Dinoflagellate abundance was positively correlated to temperature and K_d_, and was negatively correlated to salinity, NH_3_, NO_x_, PO_4_^3−^, and SiO_2_ (Table 2). Cryptophyte abundance was positively correlated to NH_3_, SiO_2_, and K_d_, and was negatively correlated to temperature, NO_x_, and PO_4_^3−^ (Table 2). Haptophyte abundance was positively correlated to K_d_, and was negatively correlated to temperature, salinity, NH_3_, NO_x_, PO_4_^3−^, and SiO_2_ (Table 2).

**Table 2.** Results for the quasipoisson and quasibinomial GLM with an offset analysis for select groups at WP and GP.

WP	Temp	Turb	NH_3_	NO_x_	PO_4_^3−^	SiO_2_	K_d_
Total diatoms	+	–	+	–		+	–
Total dinoflagellates		–		–			+
Total cryptophytes	+		+		–	+	–
Total haptophytes	+		–		+	+	+
Active mixotroph abundance	–	+	+	–		+	–
Proportion of active mixotrophs	–	+	+	–		+	–
**GP**	**Temp**	**Sal**	**NH_3_**	**NO_x_**	**PO_4_^3−^**	**SiO_2_**	**K_d_**
Total diatoms	–	+		–	–	+	+
Total dinoflagellates	+	–	–	–	–	–	+
Total cryptophytes	–		+	–	–	+	+
Total haptophytes	–	–	–	–	–	–	+
Active mixotroph abundance	–		–	–	+		+
Proportion of active mixotrophs	+	–	–	–	+	–	–

The factors temperature (Temp), turbidity (Turb), salinity (Sal), ammonium (NH_3_), nitrate + nitrite (NO_x_), phosphate (PO_4_^3−^), and silica (SiO_2_) were used in at least one of the models. (−/+): factor was positively (+) or negatively (−) related to the dependent variable. Blank space: factor did not likely have signal in the resulting GLM (*P* > .5) related to variability in the dependent variable or was removed due to collinearity (VIF > 5).

### Mixotrophic ASVs

**Figure 4.**
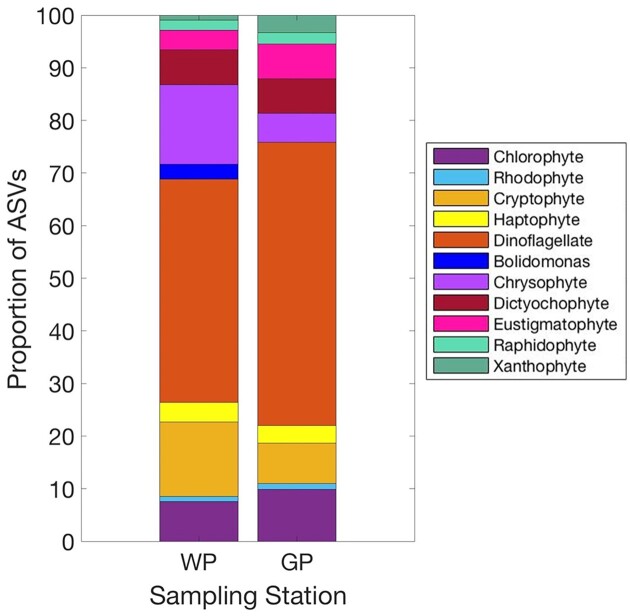
Proportion of mixotrophic ASVs of each major taxa group at (A) WP and (B) GP stations.

A total of 138 unique ASVs were identified as containing plastids and associated with the ingestion of BrdU-labeled bacteria (CMs) over the 24 sampling dates between WP and GP. A total of 109 of these ASVs were identified to at least the genus level. Out of the mixotrophic ASVs identified, 47 were unique to WP, 32 were unique to GP, and 59 occurred at both stations. Dinoflagellates ASVs made up the largest proportion (∼44%; at GP, dinoflagellates comprised ∼53% of the ASVs; Fig. 4) of all major taxa groups and were the most evenly distributed between the two stations. From the 61 dinoflagellate ASVs identified, 33 occurred at both stations, while 12 were unique to WP and 16 were unique to GP. Chrysophytes and cryptophytes were the least evenly distributed between stations. A total of 12 chrysophyte and 10 cryptophyte ASVs were unique to WP, while only one chrysophyte and two cryptophyte ASVs were unique to GP. At WP, while dinoflagellates (43%), chrysophytes (15%), and cryptophytes (14%) also accounted for a notable proportion of ASVs (Fig. 4).

**Figure 5.**
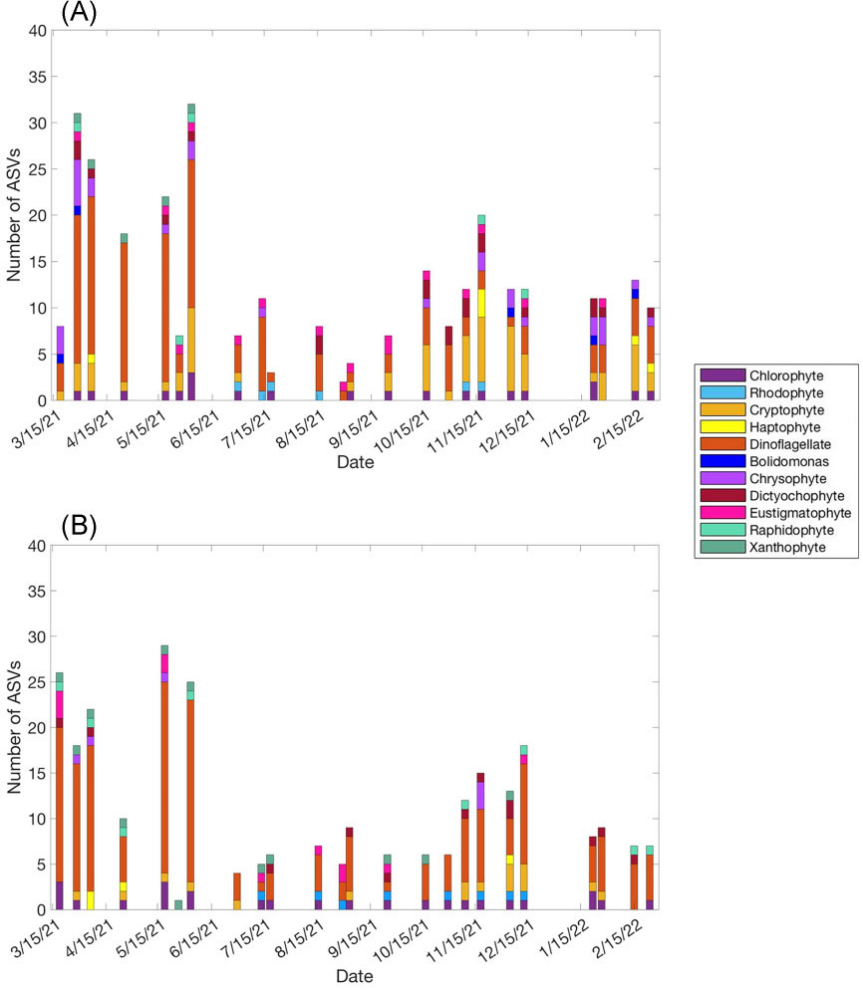
Number of active CMs for each major taxa group identified through Illumina sequencing at WP (A) and GP (B) stations for each sampling date. Each bar corresponds to the number of individual ASVs classified as being a CM based on chloroplast containing sequences that were ingesting BrdU-labeled bacteria. The *x*-axis represents a year-long timeline. Each bar represents a sampling date.

At both stations, the number of CM ASVs identified for a sampling date was highest in early spring. There was a decrease during the summer and a small increase during the autumn (Fig. 5). Dinoflagellate ASVs were the dominant mixotrophic taxa group throughout the whole year at GP. However, at WP, dinoflagellate ASVs dominated in the first half of the year with cryptophytes becoming dominant later in the year (Fig. 5). Chrysophyte ASVs were always prominent at WP, but never dominant.

**Figure 6.**
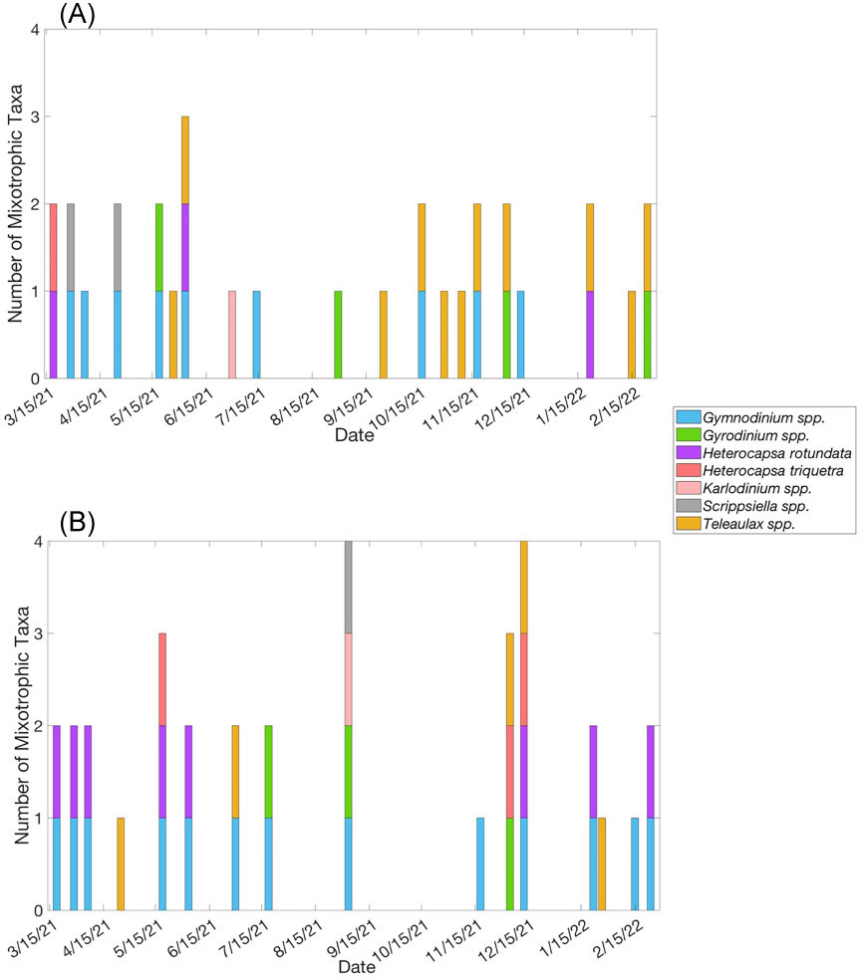
Number of genera/species of each major taxonomic group classified as an active CM in the microscopy samples at (A) WP and (B) GP stations. The *x*-axis represents a year-long timeline. Each bar represents a sampling date.

A total of 22 of the ASVs could be matched with six genera identified in microscopy samples: *Gymnodinium* spp. (*G. aureolum, G. nolleri, G. dorsalisulcum, G. palustre, G*. sp. GSSW10, *G*. uncultured eukaryote, and *G*. uncultured marine eukaryote), *Gyrodinium* spp. (*Gyrodinium* uncultured eukaryote, *G. instriatum, Gyrodinium* uncultured alveolate, *Gyrodinium* uncultured dinoflagellate, and *Gyrodinium* uncultured marine eukaryote), *Heterocapsa* spp. (*H. rotundata, H. niei, H. triquetra, Heterocapsa* uncultured dinoflagellate, and *Heterocapsa* uncultured eukaryote*), Karlodinium* sp. (*Karlodinium* uncultured marine dinoflagellate), *Scrippsiella* spp. (*Scrippsiella* sp. NY012 and *Scrippsiella* uncultured marine alveolate), and *Teleaulax* sp. (*Teleaulax* uncultured eukaryote and *Teleaulax* uncultured marine eukaryote) (Fig. 6). The remaining 116 ASVs that were identified from the BrdU experiments were not associated with taxa from the microscopy samples. Most of those taxa were either too small to be accurately identified through microscopy, or were ASVs with general identification (e.g. chrysophyceae uncultured eukaryote, dinophyceae uncultured eukaryote, and cryptophyceae uncultured freshwater eukaryote). It is also possible that a number of these taxa were too rare within the system to be captured via our light microscopy analysis.

### Potential and active CM abundance and proportion

**Figure 7.**
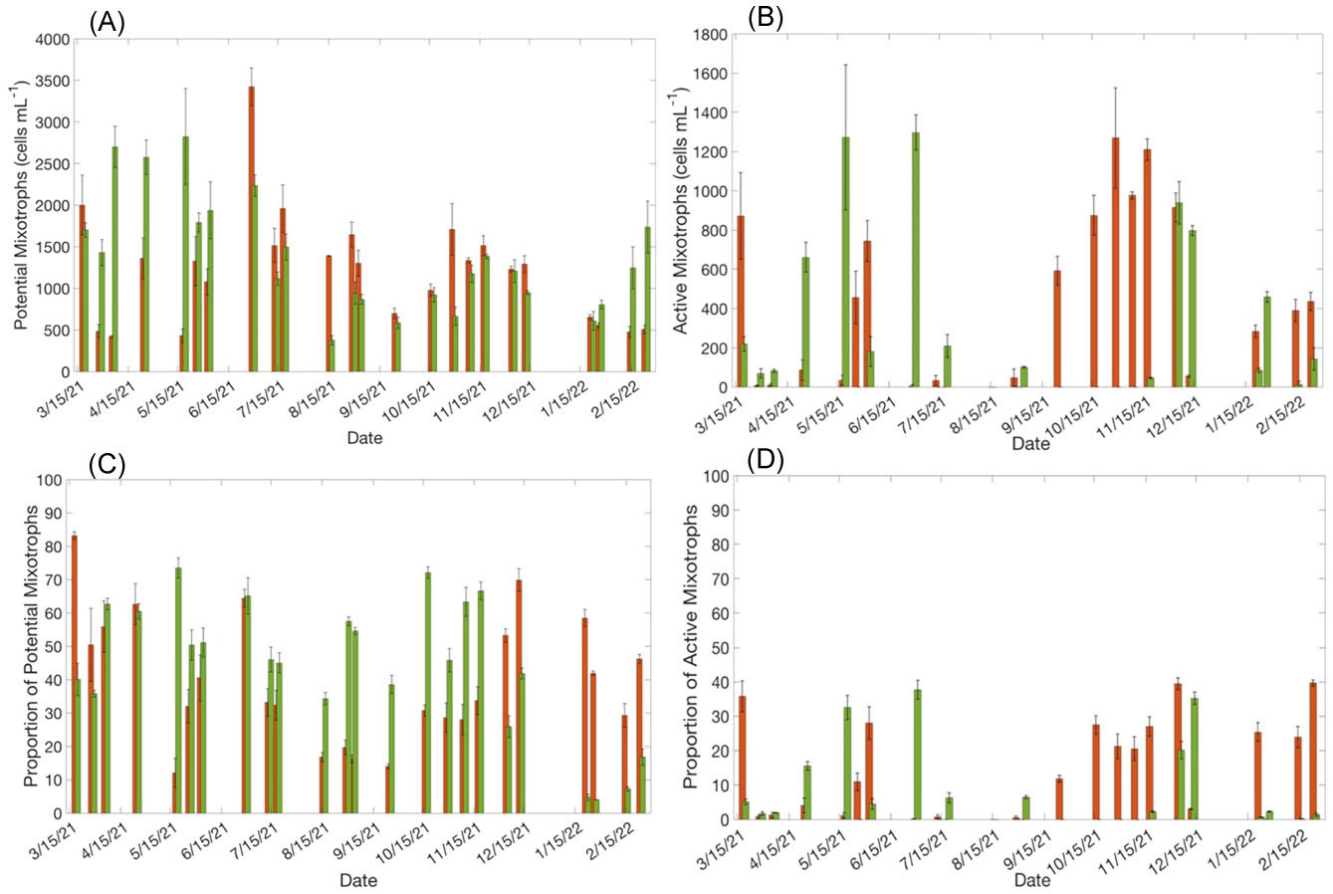
(A) Estimated abundance of potential CMs in all microscopy samples based on the genera of chloroplast-containing plankton that have demonstrated the of ingesting bacteria in previous experiments. (B) Total abundance of active CMs in microscopy samples identified through the BrdU experiments for each sampling date at WP and GP stations. The active CMs were identified based on the genera/species of chloroplast-containing plankton that were ingesting BrdU-labeled bacteria for each sampling date. (C) Estimated proportion of potential CMs in all microscopy samples based on the genera of chloroplast-containing plankton that have demonstrated the of ingesting bacteria in previous experiments. (D) The proportion of active CMs present at WP and GP stations. The proportional abundance of active CMs was calculated by dividing the abundance of genera/species identified as active CMs by the total abundance of phototrophs of each sampling date. The *x*-axis represents a year-long timeline. Each pair of bars (WP and GP) represents a single sampling date along that timeline.

At WP, the average abundance (±SE) of potential CMs was 1221 ± 132 cells ml^−1^ (Fig. 7A), which accounted for 40% ± 3.6 of the average total number of phototrophic cells counted (Fig. 7C). At GP, the average abundance of potential CMs was 1387 ± 151 cells ml^−1^ (Fig. 7A), which accounted for 44% ± 2.6 of the average proportion of total phototrophic cells counted (Fig. 7C). The abundance and proportion of potential CMs was highly variable throughout the year at both the GP (376–2824 cells ml^−1^; 4%–74%) and WP (419–3422 cells ml^−1^; 12%–83%) stations. The abundance and proportion of phototrophic cells that were potential CMs at GP was highest during spring and autumn, while at WP, abundance and proportion of phototrophic cells that were potential CMs was highest during the spring and winter.

At WP, the average abundance (±SE) of active CMs was 388 ± 56 cells ml^−1^ (Fig. 7B), which accounted for 13% ± 1.6 of the average total number of phototrophic cells counted (Fig. 7D). At GP, the average abundance of active CMs was 274 ± 41 cells ml^−1^ (Fig. 7B), which accounted for 7% ± 0.7 of the average proportion of total phototrophic cells counted (Fig. 7D). The abundance and proportion of active CMs was highly variable throughout the year at both the GP (0–1297 cells ml^−1^; 0%–38%) and WP (0–1270 cells ml^−1^; 0%–40%) stations. The abundance and proportion of phototrophic cells that were active CMs at GP was highest during summer, while at WP, abundance and proportion of phototrophic cells that were active CMs was highest during the autumn and winter.

Cryptophytes and dinoflagellates were the only two taxonomic groups represented in the potential and active CM abundance. At WP, there was seasonal variability in the dominant taxonomic group representing active CMs; during the spring, dinoflagellates such as *Gymnodinium* spp., *H. rotundata*, and *Scrippsiella* spp. dominated the community of active CMs, while the cryptophyte *Teleaulax* sp. was more frequently identified as an active CM throughout the autumn (Fig. 6A). At GP, dinoflagellate ASVs such as *Gymnodinium* spp. and *H. rotundata* were frequently identified as active CMs throughout the whole year in microscopy samples (Fig. 6B).

Based on the GLM analysis, the abundance of active CMs at WP was positively correlated to turbidity, NH_3_, and SiO_2_ concentrations, and negatively correlated to temperature, NO_x_ concentrations, and K_d_ (Table 2). The proportion of active CMs was positively correlated to turbidity, NH_3_, and SiO_2_ concentrations, and negatively correlated to temperature, NO_x_ concentrations, and K_d_ (Table 2). The abundance of active CMs at GP was positively correlated to K_d_ and PO_4_^3−^ concentrations, and negatively correlated to temperature and NH_3_ and NO_x_ concentrations (Table 2). The proportion of active CMs was positively correlated to temperature and PO_4_^3−^ concentrations, and negatively correlated to salinity, NH_3_, NO_x_, SiO_2_ concentrations, and K_d_ (Table 2).

## Discussion

The BrdU method was used to identify over 130 CMs that were actively ingesting bacteria within an estuarine system across 1 year. The BrdU data was combined with microscopy taxonomy and enumeration data to estimate the abundance and proportion of potential and active CMs that could be identified microscopically. These results demonstrated that estimations of CM abundance were higher for potential CMs compared to active CMs, suggesting that the BrdU experiments can constrain estimations of abundance when used to account for taxa known to be actively grazing in a given microscopy sample. Estimations of active CMs were further used in an assessment of what biotic and abiotic factors are associated with temporal and spatial variability of active CMs. Specifically, it was identified how the actively grazing mixotrophic taxa changed throughout the year and that there were distinct differences in the mixotrophic activity of some taxa at the different stations. The results suggested that active mixotroph abundance is not only regulated by environmental conditions generally favorable to mixotrophy but environmental conditions favorable to specific taxon’s utilization of phagotrophy.

### Potential versus active CMs analysis

Measurements of potential CM abundance compared to the abundance of active CMs were on average 37% higher at GP and 27% higher at WP (Fig. 7C). The primary reason for this difference was the presence of the cryptophyte *Teleaulax* sp. throughout the year at both stations. While *Teleaulax* sp. was continually present, it was primarily identified to be actively grazing at WP during the autumn (Fig. 6A). Estimating potential CM abundance assumes that if a taxon has the capacity to ingest prey, that it will actively be ingesting all the time. These results indicate that at least some mixotrophic taxa are likely only using their alternate trophic mode under certain conditions. Therefore, estimating the abundance of CMs based on who has the *capacity* to ingest prey can overestimate the proportion of the planktonic community that is engaged in mixotrophy at a given time. For example, a recent study that also estimated potential CM abundance and proportion in a temperate estuary (Waquoit Bay, MA, USA) using the BrdU method reported that cryptophytes were consistently a dominant part of the mixotrophic population (Millette et al. 2021). The findings of this current study suggest that Millette et al. (2021) may have overestimated the importance of cryptophytes in the CM assemblage and underestimated the importance of dinoflagellates by focusing on the *capacity* for grazing.

### Temporal and spatial variability in active CMs

On average, the abundance and proportion of CMs between the two stations was not significantly different (Fig. 7B and D). This result was not unexpected because mixotrophs are hypothesized to be dominant when one growth factor is limiting (Stoecker 1998), and each station likely had one limiting condition. The average water quality data indicated that light and nutrients were significantly different between WP and GP; light levels (K_d_) were lower at WP compared to GP, while NO_x_ was significantly higher at WP compared to GP. Average NH_3_ and PO_4_^3−^ concentrations were also higher at WP, although these differences were not significant due to high annual variability (Figures S3 and S5, Supporting Information). Overall, this aligns with what is known about the average environmental conditions at these stations, one likely being light limited for phototrophic growth (WP) and the other likely being nutrient limited for phototrophic growth (GP, Friedrichs 2009, Reay 2009). Since both stations had one likely limiting factor, it was expected that the importance of CM abundance and proportion would be equally important at both stations, albeit for different reasons.

It was, therefore, expected that the temporal variability in active CM abundance within each station would vary based on the primary growth limiting factor, light at WP and nutrients at GP. However, results from the GLM analysis were inconclusive related to these factors. At the WP station, high abundance and proportion of active CMs was associated with low nutrients (low NO_x_) and high nutrients (high NH_3_), and high light (low K_d_). This possibly suggested that actively grazing CMs are more prevalent at WP when light and nutrients are less limiting, and not negatively related to light, we hypothesized. At GP, a high abundance of active CMs was associated with both low nutrient (low NH_3_ and NO_x_) and low light (high K_d_), suggesting that actively grazing CMs are more prevalent when light and nutrients are more limiting. However, a high proportion of active CMs was associated with low nutrients (low NH_3_ and NO_x_) and high light (low K_d_), suggesting that actively grazing CMs are more prevalent when nutrients are more limiting, which is the result hypothesized. Even though the proportion of active CMs at GP was related to the one environmental factor expected, most GLM analysis results do not align with the hypothesis that CMs have an advantage when only one growth factor is limiting (Stoecker 1998, Edwards 2019, Li et al. 2023). Still, there is evidence that the differences in environmental conditions between the stations might influence the average abundance of CMs. However, further analysis suggested that factors were associated with the variability in active CM abundance at each station.

The abundance of CMs at both stations was correlated to the abundance of major taxonomic groups. At GP, the abundance of active CMs was correlated to dinoflagellate abundance (Table S3, Supporting Information). At WP, active CM abundance for the whole dataset was not correlated with any major taxa group, but active CM abundance for the first 6 months of sampling was correlated with dinoflagellate abundance and cryptophyte abundance for the second 6 months (Table S3, Supporting Information). This suggested that when more dinoflagellates were present at GP and either more dinoflagellates or cryptophytes were present at WP, depending upon the time of year, there would be more active CMs. Furthermore, the environmental patterns identified to be associated with a high abundance and proportion of CMs using GLM analyses were similar with the patterns associated with a high abundance of dinoflagellates at GP and patterns associated with high abundances of cryptophytes at WP (Table 2). This suggested that the abundance of active CMs throughout the year at a specific location was related to both the environmental conditions that favored the growth of particular taxa and each taxon’s utilization of phagotrophy. This challenges the assumption that mixotroph abundance equals mixotrophic activity and means that understanding mixotrophy on a large scale requires assessing the conditions that favor mixotrophic activity in the dominant taxon present. As with the *Teleaulax* sp. in this study, the cryptophyte was often present in the microscopy samples but only identified to be actively grazing bacteria at specific times and locations. Identifying dominant mixotrophic taxa present within a certain region and the conditions that cause them to switch nutrient modes are important to understanding how mixotrophy contributes to nutrient and carbon trophic transfer. The BrdU prey-labeling experiments used in this study provided a starting point for conducting this analysis.

### BrdU method

The BrdU method used in this study is still relatively new, and while it has potential, it also has drawbacks. First, it is costly and time-consuming compared to most basic mixotrophy detection methods. The immunoprecipitation process takes 3 days of active and precise work, and only a limited number of samples can be processed at a given time. Depending on the number of samples, the processing of samples could potentially take months. For all that effort, this method exclusively targets mixotrophs with their own chloroplasts that are ingesting bacteria (CM), excluding other types of mixotrophs. However, CMs are an important and ubiquitous mixotrophic group that commonly ingest bacteria, in addition to small protists (Jeong et al. 2010, Faure et al. 2019, Li et al. 2023, Mitra et al. 2023), which means this approach likely captures the majority of CMs present in a system. Furthermore, the BrdU method is targeting the same mixotrophs as most fluorescently labeled prey experiments, which is how most CMs are currently identified *in situ*. This method might also be biased toward the detection of mixotrophic dinoflagellates relative to other mixotrophic taxa due to their high ribosomal gene copy number (Millette et al. 2021), although a substantial number of other mixotrophic ASVs such as chlorophytes, cryptophytes, and chrysophytes were also identified. Furthermore, taxa present in high abundances such as diatoms (nonmixotrophic), might lead to nonspecific recovery of DNA, undermining confidence in species classified as mixotrophs (Flynn et al. 2013). Also, it is often difficult to microscopically identify many of the mixotrophic ASVs, potentially underestimating abundance of active CMs. However, most of the ASVs not identified in the microscopic samples consisted of taxa that were too small (< 10 µm) to be identified by microscopy and were not included in reported active CM abundances or total phototroph abundances. Therefore, the active CM abundance data should be considered a representation of CM >10–15 µm.

Although imperfect, this method identifies CM taxa in a sample actively ingesting bacteria. Thus, allowing for the creation of a list of active CMs that ingest bacteria within the environment being studied. This method not only allowed identification of over 130 CMs (80% to the genus level), but it also resulted in the creation of two separate lists of CMs unique to distinct parts of a temperate estuary along with correlation of the environmental conditions that supported their active ingestion of prey. By adjusting the sampling frequency or stations, future studies using this method could be expanded to examine the time of year or conditions that different CM ASVs are identified to be actively grazing compared to when they are present and not grazing. This would substantially improve estimations of the abundance and proportion of potential CMs from long-term microscopy-based taxonomic datasets.

## Conclusion

This study advanced our understanding of how *in situ* CM abundance and proportion are influenced by not only environmental factors, but by the taxa present. It is critical to identify active CMs, the factors favoring their abundance and proportion as active CMs, and their different responses to changes in biotic and abiotic factors (Stoecker 1998, Edwards 2019). It was demonstrated that measurements of potential CM abundance showed a substantial overestimation of the presence of CMs when compared to active CM abundance; the presence of mixotrophic taxa in the system does not necessarily mean mixotrophic activity. This highlights the limitations within studies that recategorize phytoplankton from microscopy-based taxonomic datasets to estimate mixotroph abundance, as this study shows that species were not always grazing when they were present. Analyses with historical datasets are still very useful because they can help rapidly increase understanding of large-scale mixotroph presence and distribution, but it is necessary to better constrain the organisms classified as mixotrophs based on the conditions they are grazing under. The BrdU method can be used to help identify the conditions under which CMs are actively grazing, and more accurately use those datasets. This way, our knowledge on the biogeographical distribution of CM abundance can be expanded over large time periods to better understand their contribution to aquatic food webs.

## Supplementary Material

fiae015_Supplemental_File
