# Supplementary material for: Temporal and spatial variability of constitutive mixotroph abundance and proportion
Source: FEMS Microbiol Ecol. 2024 Feb 2;100(3):fiae015. doi: 10.1093/femsec/fiae015 (PMC10939395; doi:10.1093/femsec/fiae015)
Supplement: fiae015_Supplemental_File [file fiae015_supplemental_file.docx]

**Supplement**

| **ASVs** | | |
| --- | --- | --- |
| GP | WP | BOTH |
| *Scenedesmus* sp. KMMCC 1258 | *Ostreococcus* uncultured eukaryote | *Ostreococcus* marine metagenome |
| *Monoraphidium contortum* | *Ostreococcus* uncultured marine picoeukaryote | *Amphikrikos* sp. J.C.Han 43 |
| *Mychonastes jurisii* | *Oocystaceae* sp. GSL021 | *Porphyridium sordidum* |
| *Ostreococcus tauri* | *Oocystaceae* sp. NIES 3919 | *Teleaulax* uncultured eukaryote |
| *Proteomonas* uncultured eukaryote | *Chroomonas coerulea* | *Teleaulax* uncultured marine eukaryote |
| *Algirosphaera robusta* | *Chroomonas* unidentified cryptomonad U53191 | *Cryptophyta* sp. CR-MAL11 |
| *Amphidinium* sp. HG114 | *Hemiselmis cryptochromatica* | *Katablepharis* uncultured eukaryote |
| *Amphidinium steinii* | *Hemiselmis rufescens* |  |
|  | Hemiselmis uncultured alveolate | *Isochrysis galbana* |
| *Gymnodinium palustre* | *Cryptophyta* sp. CR-MAL03 | *Chrysochromulina* uncultured marine picoeukaryote |
| *Gymnodinium* uncultured marine eukaryote | *Rhinomonas nottbecki* | *Sinophysis* uncultured marine eukaryote |
|  | *Rhodomonas baltica* | *Gymnodinium dorsalisulcum* |
| *Gymnoxanthella* uncultured eukaryote | *Chrysochromulina* uncultured Chrysochromulina | *Gymnodinium* sp. GSSW10 |
| *Lepidodinium* uncultured marine dinoflagellate | *Chrysochromulina* uncultured haptophyte | *Gymnodinium* uncultured eukaryote |
| *Pheopolykrikos hartmannii* | *Dissodinium pseudolunula* | *Lepidodinium* uncultured eukaryote |
| *Polykrikos kofoidii* | *Gymnodinium aureolum* | *Paragymnodinium* uncultured eukaryote |
| *Gyrodinium* uncultured marine eukaryote | *Gymnodinium cf. nolleri* | *Polykrikos geminatum* |
| *Akashiwo* sp. AP-LIS1 | *Paragymnodinium* uncultured marine picoplankton | *Warnowia* sp. BSL-2009a |
| *Katodinium* uncultured eukaryote | *Gymnodinium* clade uncultured alveolate | *Levanderina fissa* |
| *Suessiaceae* sp. YY1405 | *Gyrodinium* uncultured alveolate | *Gyrodinium* uncultured dinoflagellate |
| *Suessiaceae* sp. mi11-8kt | *Gyrodinium* uncultured eukaryote | *Karenia mikimotoi* |
| *Alexandrium monilatum* | *Karenia brevis* | *Karlodinium* uncultured marine dinoflagellate |
| *Ochromonas* sp. CCMP1278 | *Symbiodinium* uncultured marine plankton | *Takayama cf. pulchellum* |
| *Ciliophrys infusionum* | *Alexandrium ostenfeldii* | *Biecheleria natalensis* |
| *Pedinella* uncultured marine eukaryote | *Durinskia baltica* | *Biecheleria* uncultured alveolate |
| *Monodus subterranea* | *Scrippsiella* uncultured marine alveolate | *Biecheleria* uncultured dinoflagellate |
| *Eustigmatophyceae* sp. Itas 9/21 S-8w | *Bolidomonas* uncultured eukaryote | *Pelagodinium* uncultured eukaryote |
| *Botrydiopsis pyrenoidosa* | *Bolidomonas* uncultured stramenopile | *Pelagodinium* uncultured marine dinoflagellate |
| *Chlorellidium tetrabotrys* | *Bolidomonas* uncultured marine eukaryote | *Protodinium simplex* |
| *Trebouxiophyceae* uncultured eukaryote | *Chrysowaernella hieroglyphica* | *Symbiodinium* uncultured dinoflagellate |
| *Cryptomonadales* uncultured eukaryote | *Chrysosaccus* sp. CCMP295 | *Amphidiniopsis* uncultured eukaryote |
| *Dinophyceae* uncultured freshwater eukaryote | *Chrysocapsa* sp. UTCC280 | *Heterocapsa niei* |
| Dinoflagellata uncultured dinoflagellate | *Uroglena* uncultured *Ochromonas* sp. | *Heterocapsa triquetra* |
| Eustigmatales uncultured eukaryote | *Chromophyton vischeri* | *Heterocapsa* uncultured dinoflagellate |
|  | *Hibberdia magna* | *Heterocapsa* uncultured eukaryote |
|  | *Mallomonas* uncultured Synurales | *Scrippsiella* sp. NY012 |
|  | *Pseudopedinella elastica* | *Haplozoon* uncultured eukaryote |
|  | *Pedinellales* sp. RCC2286 | *Dinobryon faculiferum* |
|  | *Nannochloropsis* uncultured marine eukaryote | *Ochromonas* uncultured eukaryote |
|  | Cryptomonadales uncultured eukaryote | *Apedinella radians* |
|  |  | *Pseudopedinella* uncultured eukaryote |
|  | Cryptophyceae uncultured freshwater eukaryote | *Pteridomonas* uncultured eukaryote |
|  |  | *Monodus* sp. NIES-3918 |
|  | Chrysophyceae uncultured chrysophyte | *Heterosigma akashiwo* |
|  | Chrysophyceae uncultured freshwater eukaryote | Chattonellales MOCH-3 uncultured stramenopile |
|  | Chrysophyceae uncultured marine eukaryote | Mamiellophyceae uncultured eukaryote |
|  | Chrysophyceae uncultured stramenopile | Archaeplastida; Chloroplastida |
|  | Chrysophyceae uncultured marine stramenopile | Cryptomonadales uncultured cryptophyte |
|  | Pedinellales uncultured Pedinellales | Cryptomonadales uncultured marine picoeukaryote |
|  |  |  |
|  |  | *Sinophysis* uncultured dinoflagellate |
|  |  | *Suessiaceae* uncultured alveolate |
|  |  | *Suessiaceae* uncultured eukaryote |
|  |  | Dinophyceae uncultured eukaryote |
|  |  | Dinophyceae uncultured marine dinoflagellate |
|  |  | *Noctilucales* uncultured eukaryote |
|  |  | Chrysophyceae uncultured eukaryote |
|  |  | Chrysophyceae uncultured marine picoeukaryote |
|  |  | Pedinellales uncultured stramenopile |
|  |  | Eustigmatales uncultured phytoplankton |
|  |  | Eustigmatales uncultured stramenopile |
|  |  | Xanthophyceae uncultured marine eukaryote |
|  |  |  |

Table S1. List of potential mixotrophic ASVs for WP and GP stations based on phototrophic taxa were identified to be grazing at each location through the BrdU incubations.

| Vial # | Date | Station | Sample | Dilution Ratio | Vial # | Date | Station | Sample | Dilution Ratio |
| --- | --- | --- | --- | --- | --- | --- | --- | --- | --- |
| 1 | 3/19/21 | WP | +BrdU | - | 149 | 9/3/21 | WP | -BrdU | 1:50 |
| 2 | 3/19/21 | WP | +BrdU | - | 150 | 9/3/21 | WP | -BrdU | 1:50 |
| 4 | 3/19/21 | WP | -BrdU | 1:5 | 151 | 9/3/21 | GP | +BrdU | - |
| 6 | 3/19/21 | WP | -BrdU | 1:5 | 152 | 9/3/21 | GP | +BrdU | - |
| 7 | 3/19/21 | GP | +BrdU | - | 153 | 9/3/21 | GP | +BrdU | - |
| 8 | 3/19/21 | GP | +BrdU | - | 154 | 9/3/21 | GP | -BrdU | 1:20 |
| 9 | 3/19/21 | GP | +BrdU | - | 155 | 9/3/21 | GP | -BrdU | 1:20 |
| 10 | 3/19/21 | GP | -BrdU | - | 156 | 9/3/21 | GP | -BrdU | 1:20 |
| 11 | 3/19/21 | GP | -BrdU | 1:50 | 157 | 9/25/21 | WP | +BrdU | - |
| 12 | 3/19/21 | GP | -BrdU | - | 158 | 9/25/21 | WP | +BrdU | - |
| 13 | 3/29/21 | WP | +BrdU | - | 159 | 9/25/21 | WP | +BrdU | - |
| 14 | 3/29/21 | WP | +BrdU | - | 160 | 9/25/21 | WP | -BrdU | 1:20 |
| 15 | 3/29/21 | WP | +BrdU | - | 161 | 9/25/21 | WP | -BrdU | 1:20 |
| 16 | 3/29/21 | WP | -BrdU | 1:50 | 162 | 9/25/21 | WP | -BrdU | 1:20 |
| 17 | 3/29/21 | WP | -BrdU | 1:50 | 163 | 9/25/21 | GP | +BrdU | - |
| 19 | 3/29/21 | GP | +BrdU | - | 164 | 9/25/21 | GP | +BrdU | - |
| 21 | 3/29/21 | GP | +BrdU | - | 165 | 9/25/21 | GP | +BrdU | - |
| 23 | 3/29/21 | GP | -BrdU | - | 166 | 9/25/21 | GP | -BrdU | 1:20 |
| 24 | 3/29/21 | GP | -BrdU | 1:10 | 167 | 9/25/21 | GP | -BrdU | 1:20 |
| 25 | 4/6/21 | WP | +BrdU | - | 168 | 9/25/21 | GP | -BrdU | 1:20 |
| 27 | 4/6/21 | WP | +BrdU | - | 169 | 10/17/21 | WP | +BrdU | - |
| 28 | 4/6/21 | WP | -BrdU | 1:50 | 170 | 10/17/21 | WP | +BrdU | - |
| 29 | 4/6/21 | WP | -BrdU | 1:50 | 171 | 10/17/21 | WP | +BrdU | - |
| 31 | 4/6/21 | GP | +BrdU | - | 172 | 10/17/21 | WP | -BrdU | 1:20 |
| 32 | 4/6/21 | GP | +BrdU | - | 173 | 10/17/21 | WP | -BrdU | 1:20 |
| 33 | 4/6/21 | GP | +BrdU | - | 174 | 10/17/21 | WP | -BrdU | 1:20 |
| 34 | 4/6/21 | GP | -BrdU | - | 175 | 10/17/21 | GP | +BrdU | - |
| 35 | 4/6/21 | GP | -BrdU | - | 176 | 10/17/21 | GP | +BrdU | - |
| 36 | 4/6/21 | GP | -BrdU | 1:50 | 177 | 10/17/21 | GP | +BrdU | - |
| 37 | 4/25/21 | WP | +BrdU | - | 178 | 10/17/21 | GP | -BrdU | 1:20 |
| 38 | 4/25/21 | WP | +BrdU | - | 179 | 10/17/21 | GP | -BrdU | 1:20 |
| 39 | 4/25/21 | WP | +BrdU | - | 180 | 10/17/21 | GP | -BrdU | 1:20 |
| 40 | 4/25/21 | WP | -BrdU | 1:50 | 181 | 10/30/21 | WP | +BrdU | - |
| 41 | 4/25/21 | WP | -BrdU | 1:50 | 182 | 10/30/21 | WP | +BrdU | - |
| 42 | 4/25/21 | WP | -BrdU | 1:50 | 183 | 10/30/21 | WP | +BrdU | - |
| 43 | 4/25/21 | GP | +BrdU | - | 184 | 10/30/21 | WP | -BrdU | 1:20 |
| 44 | 4/25/21 | GP | +BrdU | - | 185 | 10/30/21 | WP | -BrdU | 1:20 |
| 45 | 4/25/21 | GP | +BrdU | - | 186 | 10/30/21 | WP | -BrdU | 1:20 |
| 46 | 4/25/21 | GP | -BrdU | 1:50 | 187 | 10/30/21 | GP | +BrdU | - |
| 47 | 4/25/21 | GP | -BrdU | 1:50 | 188 | 10/30/21 | GP | +BrdU | - |
| 48 | 4/25/21 | GP | -BrdU | 1:50 | 189 | 10/30/21 | GP | +BrdU | - |
| 49 | 5/19/21 | WP | +BrdU | - | 190 | 10/30/21 | GP | -BrdU | 1:20 |
| 51 | 5/19/21 | WP | +BrdU | - | 191 | 10/30/21 | GP | -BrdU | 1:20 |
| 52 | 5/19/21 | WP | -BrdU | 1:50 | 192 | 10/30/21 | GP | -BrdU | 1:20 |
| 53 | 5/19/21 | WP | -BrdU | 1:10 | 193 | 11/9/21 | WP | +BrdU | - |
| 54 | 5/19/21 | WP | -BrdU | 1:10 | 194 | 11/9/21 | WP | +BrdU | - |
| 55 | 5/19/21 | GP | +BrdU | - | 195 | 11/9/21 | WP | +BrdU | - |
| 56 | 5/19/21 | GP | +BrdU | - | 196 | 11/9/21 | WP | -BrdU | 1:20 |
| 57 | 5/19/21 | GP | +BrdU | - | 197 | 11/9/21 | WP | -BrdU | 1:20 |
| 58 | 5/19/21 | GP | -BrdU | 1:100 | 198 | 11/9/21 | WP | -BrdU | 1:20 |
| 59 | 5/19/21 | GP | -BrdU | 1:50 | 199 | 11/9/21 | GP | +BrdU | - |
| 60 | 5/19/21 | GP | -BrdU | 1:50 | 200 | 11/9/21 | GP | +BrdU | - |
| 61 | 5/27/21 | WP | +BrdU | - | 201 | 11/9/21 | GP | +BrdU | - |
| 62 | 5/27/21 | WP | +BrdU | - | 202 | 11/9/21 | GP | -BrdU | 1:20 |
| 63 | 5/27/21 | WP | +BrdU | - | 203 | 11/9/21 | GP | -BrdU | 1:20 |
| 64 | 5/27/21 | WP | -BrdU | 1:50 | 204 | 11/9/21 | GP | -BrdU | 1:20 |
| 65 | 5/27/21 | WP | -BrdU | 1:50 | 205 | 11/18/21 | WP | +BrdU | - |
| 66 | 5/27/21 | WP | -BrdU | 1:50 | 206 | 11/18/21 | WP | +BrdU | - |
| 67 | 5/27/21 | GP | +BrdU | - | 207 | 11/18/21 | WP | +BrdU | - |
| 68 | 5/27/21 | GP | +BrdU | - | 208 | 11/18/21 | WP | -BrdU | 1:20 |
| 69 | 5/27/21 | GP | +BrdU | - | 209 | 11/18/21 | WP | -BrdU | 1:20 |
| 70 | 5/27/21 | GP | -BrdU | 1:50 | 210 | 11/18/21 | WP | -BrdU | 1:20 |
| 71 | 5/27/21 | GP | -BrdU | 1:50 | 211 | 11/18/21 | GP | +BrdU | - |
| 72 | 5/27/21 | GP | -BrdU | 1:50 | 212 | 11/18/21 | GP | +BrdU | - |
| 73 | 6/3/21 | WP | +BrdU | - | 213 | 11/18/21 | GP | +BrdU | - |
| 74 | 6/3/21 | WP | +BrdU | - | 214 | 11/18/21 | GP | -BrdU | 1:20 |
| 75 | 6/3/21 | WP | +BrdU | - | 215 | 11/18/21 | GP | -BrdU | 1:20 |
| 76 | 6/3/21 | WP | -BrdU | 1:50 | 216 | 11/18/21 | GP | -BrdU | 1:20 |
| 77 | 6/3/21 | WP | -BrdU | 1:50 | 217 | 12/5/21 | WP | +BrdU | - |
| 78 | 6/3/21 | WP | -BrdU | - | 218 | 12/5/21 | WP | +BrdU | - |
| 79 | 6/3/21 | GP | +BrdU | - | 219 | 12/5/21 | WP | +BrdU | - |
| 80 | 6/3/21 | GP | +BrdU | - | 220 | 12/5/21 | WP | -BrdU | 1:20 |
| 81 | 6/3/21 | GP | +BrdU | - | 221 | 12/5/21 | WP | -BrdU | 1:20 |
| 82 | 6/3/21 | GP | -BrdU | 1:50 | 222 | 12/5/21 | WP | -BrdU | 1:20 |
| 83 | 6/3/21 | GP | -BrdU | 1:10 | 223 | 12/5/21 | GP | +BrdU | - |
| 84 | 6/3/21 | GP | -BrdU | 1:50 | 224 | 12/5/21 | GP | +BrdU | - |
| 85 | 6/30/21 | WP | +BrdU | - | 225 | 12/5/21 | GP | +BrdU | - |
| 86 | 6/30/21 | WP | +BrdU | - | 226 | 12/5/21 | GP | -BrdU | 1:20 |
| 87 | 6/30/21 | WP | +BrdU | - | 227 | 12/5/21 | GP | -BrdU | 1:20 |
| 88 | 6/30/21 | WP | -BrdU | 1:10 | 228 | 12/5/21 | GP | -BrdU | 1:20 |
| 89 | 6/30/21 | WP | -BrdU | 1:10 | 229 | 12/13/21 | WP | +BrdU | - |
| 90 | 6/30/21 | WP | -BrdU | 1:10 | 230 | 12/13/21 | WP | +BrdU | - |
| 91 | 6/30/21 | GP | +BrdU | - | 231 | 12/13/21 | WP | +BrdU | - |
| 92 | 6/30/21 | GP | +BrdU | - | 232 | 12/13/21 | WP | -BrdU | 1:20 |
| 93 | 6/30/21 | GP | +BrdU | - | 233 | 12/13/21 | WP | -BrdU | 1:20 |
| 94 | 6/30/21 | GP | -BrdU | 1:20 | 234 | 12/13/21 | WP | -BrdU | 1:20 |
| 95 | 6/30/21 | GP | -BrdU | 1:20 | 235 | 12/13/21 | GP | +BrdU | - |
| 96 | 6/30/21 | GP | -BrdU | 1:20 | 236 | 12/13/21 | GP | +BrdU | - |
| 97 | 7/14/21 | WP | +BrdU | - | 237 | 12/13/21 | GP | +BrdU | - |
| 98 | 7/14/21 | WP | +BrdU | - | 238 | 12/13/21 | GP | -BrdU | 1:20 |
| 99 | 7/14/21 | WP | +BrdU | - | 239 | 12/13/21 | GP | -BrdU | 1:20 |
| 100 | 7/14/21 | WP | -BrdU | 1:10 | 240 | 12/13/21 | GP | -BrdU | 1:20 |
| 101 | 7/14/21 | WP | -BrdU | 1:10 | 241 | 1/22/22 | WP | +BrdU | - |
| 102 | 7/14/21 | WP | -BrdU | 1:10 | 242 | 1/22/22 | WP | +BrdU | - |
| 103 | 7/14/21 | GP | +BrdU | - | 243 | 1/22/22 | WP | +BrdU | - |
| 104 | 7/14/21 | GP | +BrdU | - | 244 | 1/22/22 | WP | -BrdU | 1:20 |
| 105 | 7/14/21 | GP | +BrdU | - | 245 | 1/22/22 | WP | -BrdU | 1:20 |
| 106 | 7/14/21 | GP | -BrdU | 1:20 | 246 | 1/22/22 | WP | -BrdU | 1:20 |
| 107 | 7/14/21 | GP | -BrdU | 1:20 | 247 | 1/22/22 | GP | +BrdU | - |
| 108 | 7/14/21 | GP | -BrdU | 1:20 | 248 | 1/22/22 | GP | +BrdU | - |
| 109 | 7/19/21 | WP | +BrdU | - | 249 | 1/22/22 | GP | +BrdU | - |
| 110 | 7/19/21 | WP | +BrdU | - | 250 | 1/22/22 | GP | -BrdU | 1:20 |
| 111 | 7/19/21 | WP | +BrdU | - | 251 | 1/22/22 | GP | -BrdU | 1:20 |
| 112 | 7/19/21 | WP | -BrdU | 1:10 | 252 | 1/22/22 | GP | -BrdU | 1:20 |
| 113 | 7/19/21 | WP | -BrdU | 1:10 | 253 | 1/27/22 | WP | +BrdU | - |
| 114 | 7/19/21 | WP | -BrdU | 1:20 | 254 | 1/27/22 | WP | +BrdU | - |
| 115 | 7/19/21 | GP | +BrdU | - | 255 | 1/27/22 | WP | +BrdU | - |
| 116 | 7/19/21 | GP | +BrdU | - | 256 | 1/27/22 | WP | -BrdU | 1:50 |
| 117 | 7/19/21 | GP | +BrdU | - | 257 | 1/27/22 | WP | -BrdU | 1:20 |
| 118 | 7/19/21 | GP | -BrdU | 1:20 | 258 | 1/27/22 | WP | -BrdU | 1:20 |
| 119 | 7/19/21 | GP | -BrdU | 1:20 | 259 | 1/27/22 | GP | +BrdU | - |
| 120 | 7/19/21 | GP | -BrdU | 1:20 | 260 | 1/27/22 | GP | +BrdU | - |
| 121 | 8/16/21 | WP | +BrdU | - | 261 | 1/27/22 | GP | +BrdU | - |
| 122 | 8/16/21 | WP | +BrdU | - | 262 | 1/27/22 | GP | -BrdU | 1:20 |
| 123 | 8/16/21 | WP | +BrdU | - | 263 | 1/27/22 | GP | -BrdU | 1:20 |
| 124 | 8/16/21 | WP | -BrdU | 1:50 | 264 | 1/27/22 | GP | -BrdU | 1:20 |
| 125 | 8/16/21 | WP | -BrdU | 1:50 | 265 | 2/15/22 | WP | +BrdU | - |
| 126 | 8/16/21 | WP | -BrdU | 1:50 | 266 | 2/15/22 | WP | +BrdU | - |
| 127 | 8/16/21 | GP | +BrdU | - | 267 | 2/15/22 | WP | +BrdU | - |
| 128 | 8/16/21 | GP | +BrdU | - | 268 | 2/15/22 | WP | -BrdU | 1:20 |
| 129 | 8/16/21 | GP | +BrdU | - | 269 | 2/15/22 | WP | -BrdU | 1:20 |
| 130 | 8/16/21 | GP | -BrdU | 1:20 | 270 | 2/15/22 | WP | -BrdU | 1:20 |
| 131 | 8/16/21 | GP | -BrdU | 1:20 | 271 | 2/15/22 | GP | +BrdU | - |
| 132 | 8/16/21 | GP | -BrdU | 1:20 | 272 | 2/15/22 | GP | +BrdU | - |
| 133 | 8/30/21 | WP | +BrdU | - | 273 | 2/15/22 | GP | +BrdU | - |
| 134 | 8/30/21 | WP | +BrdU | - | 274 | 2/15/22 | GP | -BrdU | 1:20 |
| 135 | 8/30/21 | WP | +BrdU | - | 275 | 2/15/22 | GP | -BrdU | 1:50 |
| 136 | 8/30/21 | WP | -BrdU | 1:20 | 276 | 2/15/22 | GP | -BrdU | 1:20 |
| 137 | 8/30/21 | WP | -BrdU | 1:20 | 277 | 2/24/22 | WP | +BrdU | - |
| 138 | 8/30/21 | WP | -BrdU | 1:20 | 278 | 2/24/22 | WP | +BrdU | - |
| 139 | 8/30/21 | GP | +BrdU | - | 279 | 2/24/22 | WP | +BrdU | - |
| 140 | 8/30/21 | GP | +BrdU | - | 280 | 2/24/22 | WP | -BrdU | 1:20 |
| 141 | 8/30/21 | GP | +BrdU | - | 281 | 2/24/22 | WP | -BrdU | 1:20 |
| 142 | 8/30/21 | GP | -BrdU | 1:100 | 282 | 2/24/22 | WP | -BrdU | 1:20 |
| 143 | 8/30/21 | GP | -BrdU | 1:20 | 283 | 2/24/22 | GP | +BrdU | - |
| 144 | 8/30/21 | GP | -BrdU | 1:20 | 284 | 2/24/22 | GP | +BrdU | - |
| 145 | 9/3/21 | WP | +BrdU | - | 285 | 2/24/22 | GP | +BrdU | - |
| 146 | 9/3/21 | WP | +BrdU | - | 286 | 2/24/22 | GP | -BrdU | 1:20 |
| 147 | 9/3/21 | WP | +BrdU | - | 287 | 2/24/22 | GP | -BrdU | 1:20 |
| 148 | 9/3/21 | WP | -BrdU | 1:50 | 288 | 2/24/22 | GP | -BrdU | 1:20 |

Table S2. Dilution factors for samples amplified. Dashes (-) represent samples that did not need to be diluted for amplification.

| Station | Taxa Group | Correlation | |
| --- | --- | --- | --- |
|  |  | First Half of Sampling Year | Second Half of Sampling Year |
| WP | Dinoflagellates | 0.3 |  |
|  | Cryptophytes |  | 0.6 |
| GP | Dinoflagellates | 0.5 | |

Table S3. Significant correlation (p < 0.05) between the abundance of active CMs at both WP and GP stations and the abundance of major taxonomic groups. Blank space: abundance of major taxonomic group was not correlated to the abundance of CMs.


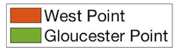

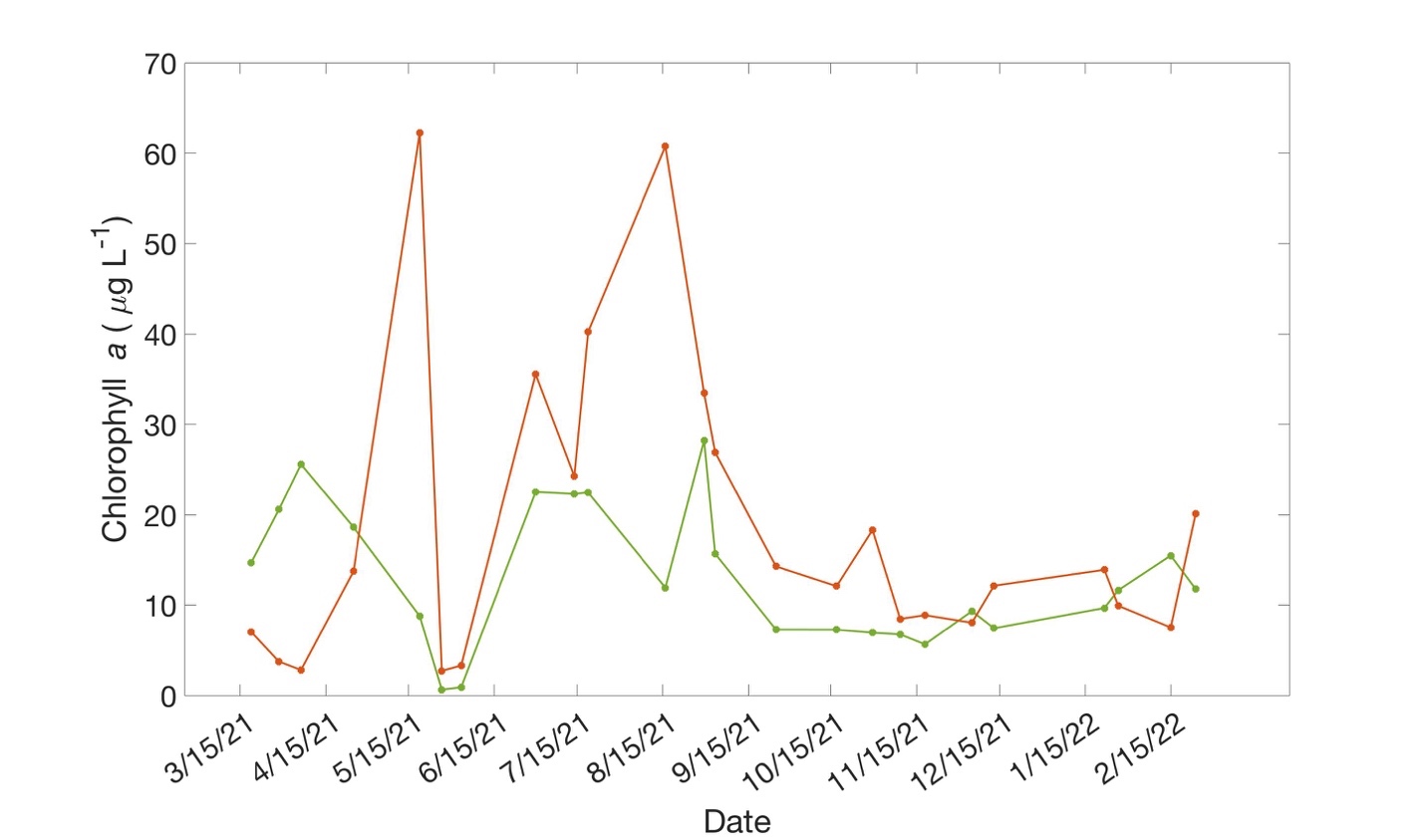


Figure S1. Time series of chlorophyll *a* measurements at 0.5m for both WP and GP stations.


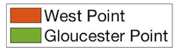

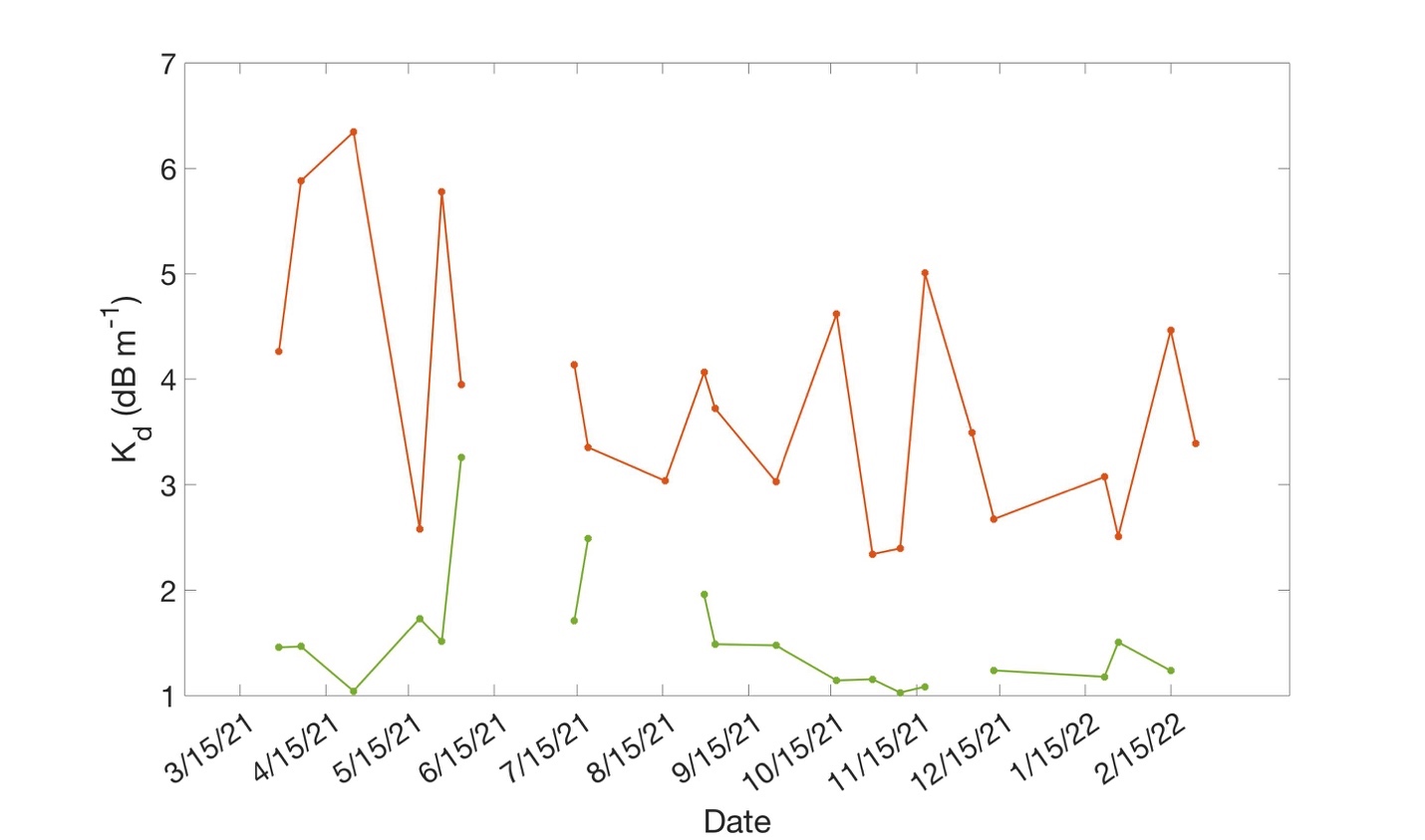


Figure S2. Time series of K_d_ measurements at 0.5m for both WP and GP stations.


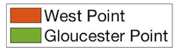

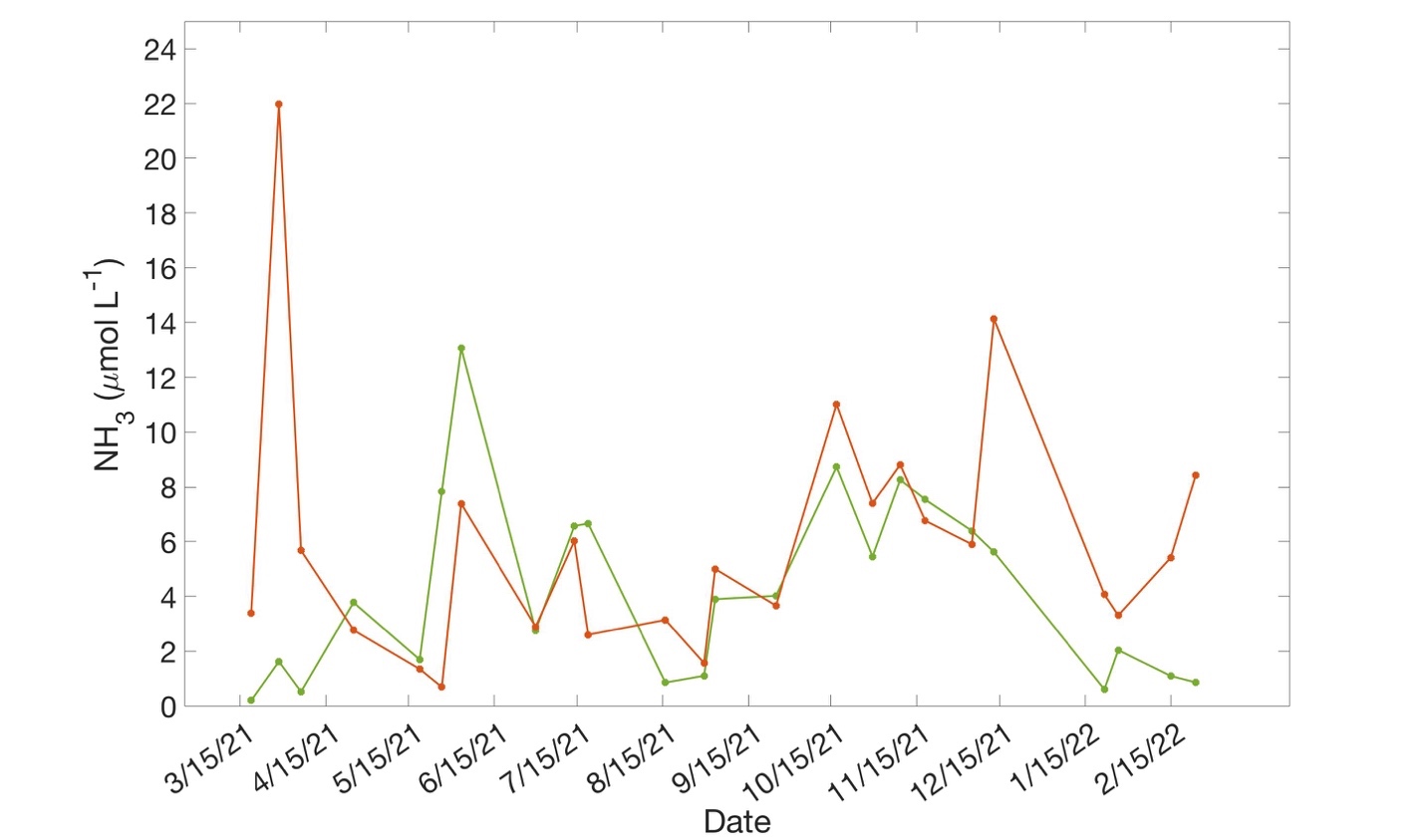


Figure S3. Time series of NH_3_ measurements at 0.5m for both WP and GP stations.


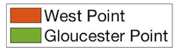

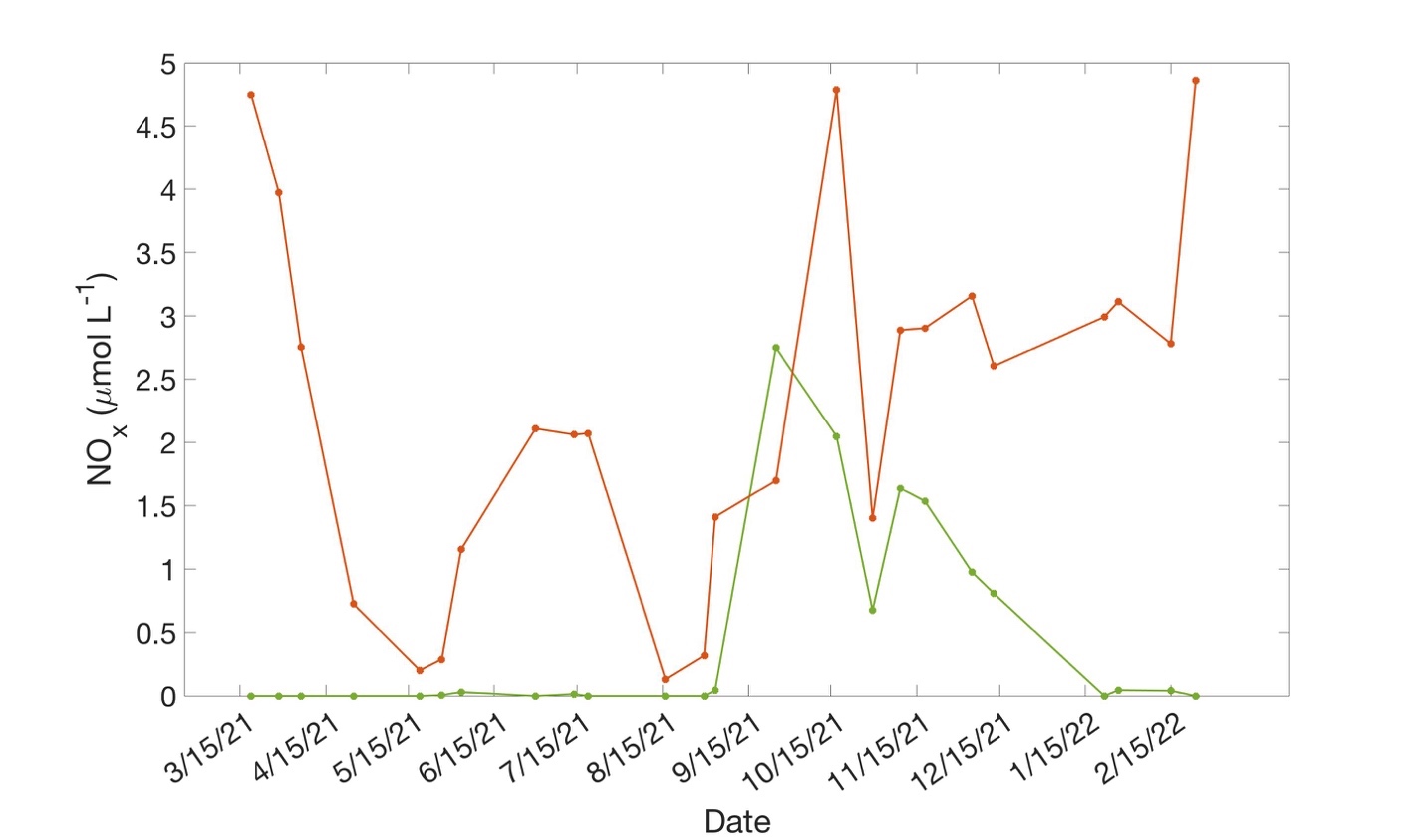


Figure S4. Time series of NO_x_ measurements at 0.5m for both WP and GP stations.


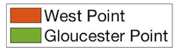

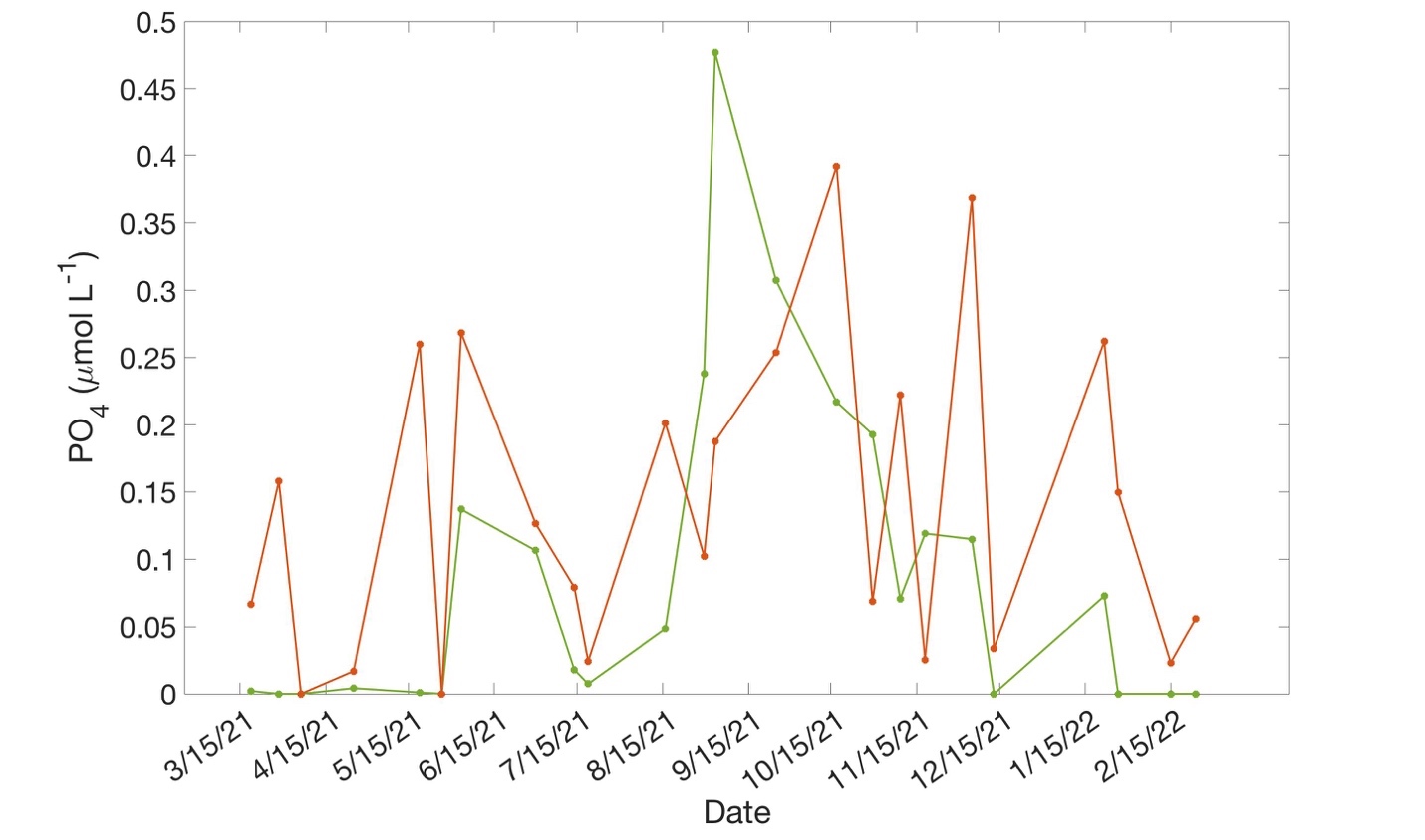


Figure S5. Time series of PO_4_ measurements at 0.5m for both WP and GP stations.


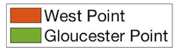

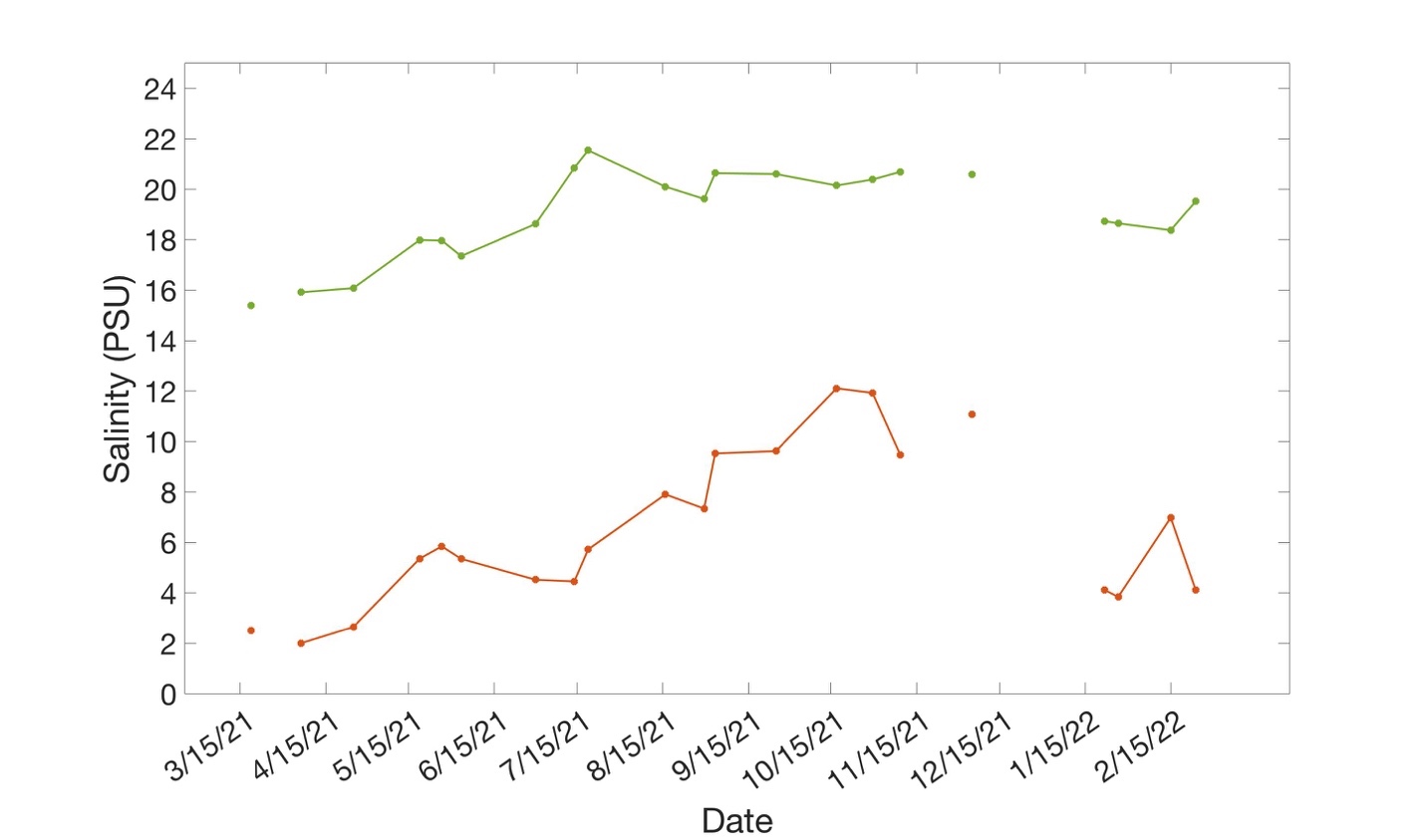


Figure S6. Time series of salinity measurements at 0.5m for both WP and GP stations.


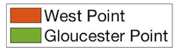

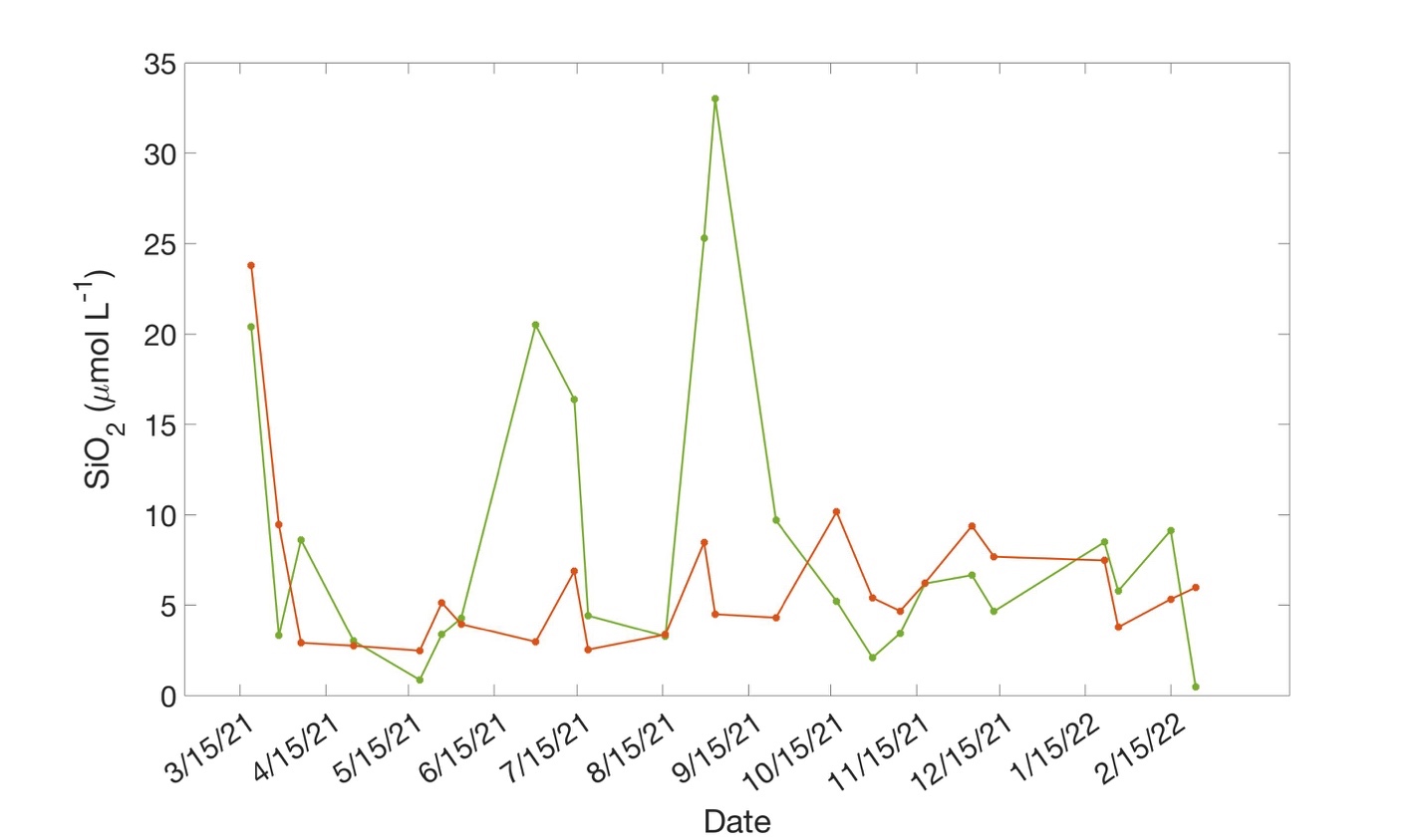


Figure S7. Time series of SiO_2_ measurements at 0.5m for both WP and GP stations.


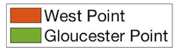

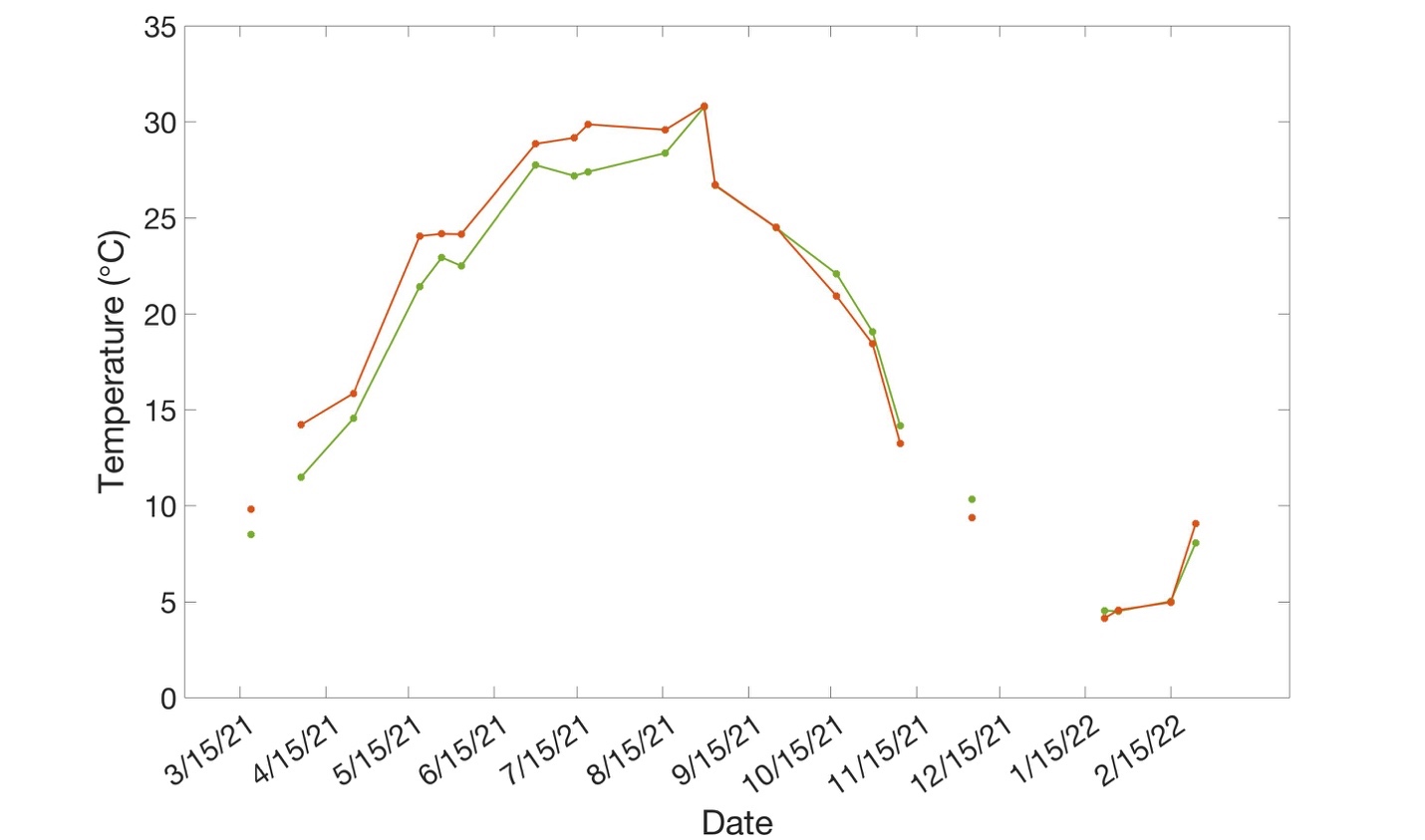


Figure S8. Time series of temperature measurements at 0.5m for both WP and GP stations.


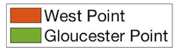

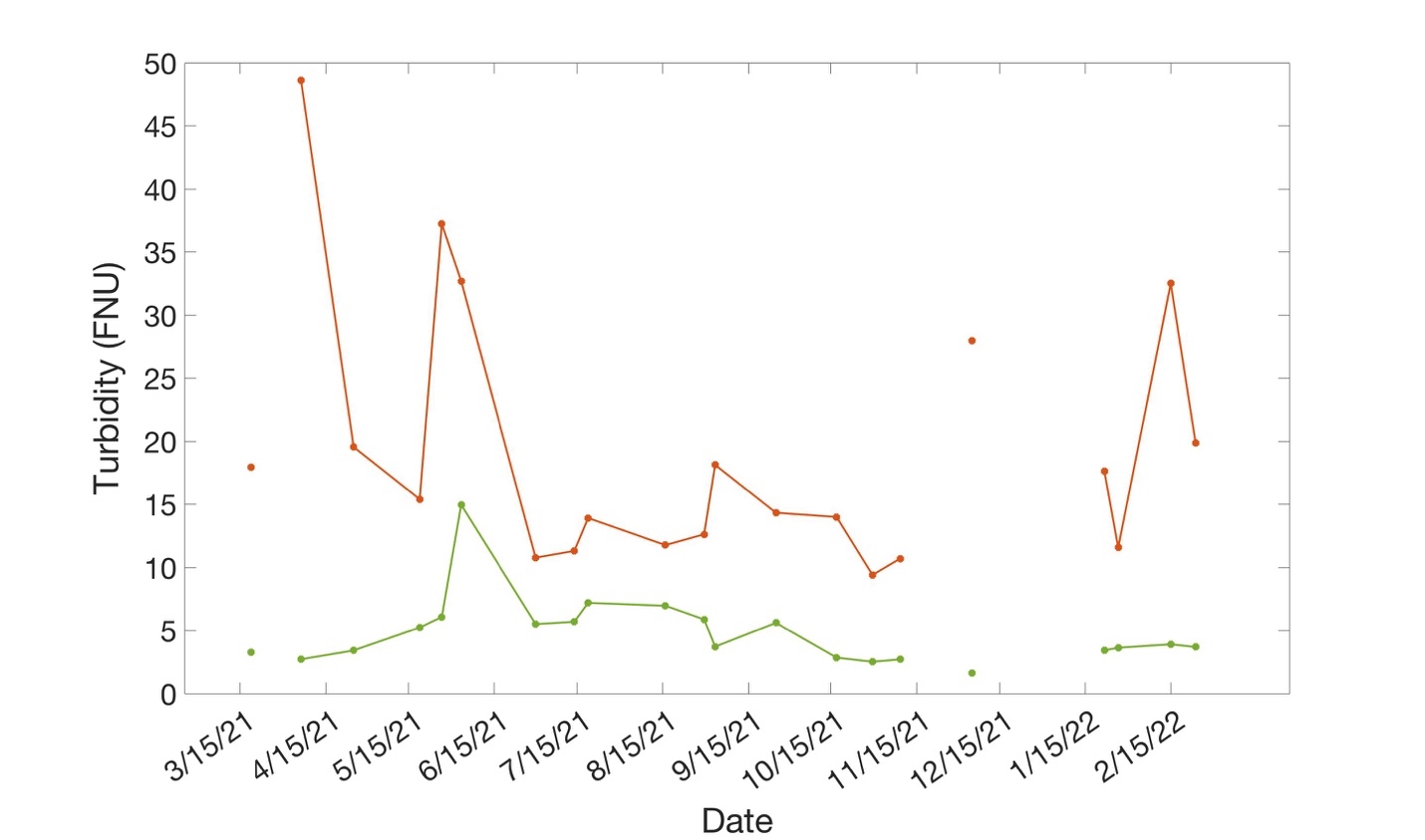


Figure S9. Time series of turbidity measurements at 0.5m for both WP and GP stations.

**Amplicon sequence**

These are the basic qiime2 steps that were used to analyze my MiSeq data.

*Demultiplex*

qiime tools import --type ‘SampleData[PairedEndSequencesWithQuality]’ --input-path <manifest file name> --output-path <name.qza> --input-format SingleEndFastqManifestPhred33V2

*Denoise, dereplicate, quality filter & remove chimeras*

qiime dada2 denoise-paired --i-demultiplexed-seqs <.qza file from above> --p-trim-left-f 10 --p-trim-left-r 10 --p-trunc-len-f 290 --p-trunc-len-r 155 --p-max-ee-f 10 --p-max-ee-r 10 --p-chimera-method pooled --o-table <name.qza> --o-representative-sequences <name.qza> --o-denoising-stats <name.qza>

*Merge table and repseq files*

qiime feature-table merge --i-tables <list tables> --o-merged-table <new table name> qiime feature-table merge-seqs --i-data <list of repset files> --o-merged-data <new file name>

*Cluster sequences based upon identity*

qiime vsearch cluster-features-de-novo --i-table <name.qza> (table created by dada2) --i- sequences <name.qza> (rep-seqs file created by dada2) --p-perc-identity 1.0 --o-clustered- table <name.qza> --o-clustered-sequences <name.qza>

*Remove singletons*

qiime feature-table filter-features --i-table <name of table generated in prior step> --p-min-frequency 3 --o-filtered-table <new table name .qza>

qiime feature-table filter-seqs --i-data <name of rep seq file created in prior step> --i-table <name of filtered table> --o-filtered-data <new repseq file .qza>

*Assign taxonomy*

qiime feature-classifier classify-consensus-vsearch --i-query <name.qza> (this is the rep-set of sequences just created by cluster) --i-reference-reads silva132_99.qza --i-reference- taxonomy taxonomy_all_levels.qza --p-maxaccepts 1 --o-classification <name.qza>
